# A Comparison Study on Criteria to Select the Most Adequate Weighting Matrix

**DOI:** 10.3390/e21020160

**Published:** 2019-02-08

**Authors:** Marcos Herrera, Jesus Mur, Manuel Ruiz

**Affiliations:** 1 CONICET-IELDE, National University of Salta, Salta A4408FVY, Argentina; 2 Department of Economic Analysis, University of Zaragoza, 50005 Zaragoza, Spain; 3 Department of Quantitative Methods, Law and Modern Languages, Technical University of Cartagena, 30201 Cartagena, Spain

**Keywords:** weights matrix, model selection, entropy, Monte Carlo

## Abstract

The practice of spatial econometrics revolves around a weighting matrix, which is often supplied by the user on previous knowledge. This is the so-called W issue. Probably, the aprioristic approach is not the best solution although, presently, there are few alternatives for the user. Our contribution focuses on the problem of selecting a W matrix from among a finite set of matrices, all of them considered appropriate for the case. We develop a new and simple method based on the entropy corresponding to the distribution of probability estimated for the data. Other alternatives, which are common in current applied work, are also reviewed. The paper includes a large study of Monte Carlo to calibrate the effectiveness of our approach compared to others. A well-known case study is also included.

## 1. Introduction

Let us begin with a mantra: the weighting matrix, usually denoted by **W**, is the most characteristic element in a spatial model. Most scholars agree with this commonplace. In fact, spatial models deal primarily with phenomena such as spillovers, trans-boundary competition or cooperation, flows of trade, migration, knowledge, etc. in complex networks. Rarely does the user know about how these events operate in practice. Indeed, they are mostly unobservable phenomena which are, however, required to build the model. Traditionally the gap has been solved by providing externally this information, in the form of a weighting matrix. As an additional remark, we should note that W is not the unique solution to deal with such kind of unobservables ([1]; for example, develop a latent-variables approach that does not need W), but is the simplest.

Hays et al. [2] give a sensible explanation about the preference for a predefined W. Network analysts are more interested in the formation of networks, taking units attributes and behaviors as given. This is spatial dependence due to selection, where relations of homophily and heterophily are crucial. The spatial econometricians are more interested in what they call *“computing the effects of alters actions on ego’s actions through the network*”; in this case, the patterns of connectivity are taken as given. This form of spatial dependence is due to the influence between the individuals, and the notions of contagion and interdependence are capital. Therefore, if the network is predefined, why not supply it externally?

However, beyond this point, the W issue has been frequent cause of dispute. In the early stages, terms such as “join” or “link” were very common (for instance, in [3], or [4]). The focus at that time was mainly on testing for the presence of spatial effects, for which is not so important the specification of a very detailed weighting matrix; contiguity, nearness, rough measures of separation may be appropriate notions for that purpose. The work of Ord [5] is a milestone in the evolution of this issue because of its strong emphasis on the task of modelling spatial relationships. It is evident that the weights matrix needs more attention if we want to avoid estimation biases and wrong inference. Anselin [6] and Anselin [7] puts the W matrix in the center of the debate about specification of spatial models, but, after decades of practicing, the question remains unclear.

The purpose of the so-called W is to “*determine which ... units in the spatial system have an influence on the particular unit under consideration ... expressed in notions of neighborhood and nearest-neighbor*” relations, in words of Anselin [6] (p.16) or “*to define for any set of points or area objects the spatial relationships that exist between them*” as stated by Haining [8] (p. 74). The problem is how should it be done.

Roughly speaking, we may distinguish two approaches: (i) specifying W exogenously; (ii) estimating W from data. The exogenous approach is by far the most popular and includes, for example, use of a binary contiguity criterion, k-nearest neighbors, kernel functions based on distance, etc. The second approach uses the topology of the space and the nature of the data, and takes many forms. We find ad-hoc procedures in which a predefined objective guides the search such as the maximization of Moran’s *I* in Kooijman [9] or the local statistical model of Getis and Aldstadt [10]. Benjanuvatra and Burridge [11] develop a quasi-maximum-likelihood, QML, algorithm to estimate the weights in W assuming partial knowledge about the form of the weights. More flexible approaches are possible if we have panel information such as in Bhattacharjee and Jensen-Butler [12] or Beenstock and Felsenstein [13]. Endogeneity of the weight matrix is another topic introduced recently in the field (i.e., [14]), which connects with the concept of *coevolution* put forward by Snijders et al. [15] and based on the assumption that in the long run, network connectivity must evolve endogenously with the model. Indeed, much of the recent literature on spatial econometrics revolves around endogeneity, but our contribution pertains to the exogenous approach where remains most part of the applied research.

Before continuing, we may wonder if the W issue, even in our context of pure exogeneity, is really a problem to worry for or it is the *biggest myth* of the discipline as claimed by LeSage and Pace [16]. Their argument is that only dramatic different choices for W would lead to significant differences in the estimates or in the inference. We partly agree with them in the sense that is a bit silly to argue whether it is better the 5 or the 6 nearest-neighbor matrix; surely there will be almost no difference between the two. However, there is consistent evidence, obtained mainly by studies of Monte Carlo [17,18,19,20] showing that the misspecification of W has a non-negligible impact on the inference of the coefficients of spatial dependence and other terms in the model. Moreover, the magnitude of the bias increases for the estimates of the marginal direct/indirect effects. Therefore, we disagree with the notion that ’*far too much effort has gone into fine-tuning spatial weight matrices*’ as stated by LeSage and Pace [16]. Our impression is that any useful result should be welcomed in this field and, especially, we need practical, clear guides to approach the problem.

Another question of concern are the criticisms of Gibbons and Overman [21]. As said, it is common in spatial econometrics to assume that the weighting matrix is known, which is the cause of identification problems; this flaw extends to the instruments, moment conditions, etc. There is little to say in relation to this point. In fact, spatial parameters (i.e., ρ) and weighting matrix, W, are only jointly identified (we do estimate ρW). Hays et al. [2] and Bhattacharjee and Jensen-Butler [12] agree on this point.

Bavaud [22] (p. 153), given this controversial debate, was very skeptical, “*there is no such thing as “true”, “universal” spatial weights, optimal in all situations*’ and continues by stating that the weighting matrix ’*must reflect the properties of the particular phenomena, properties which are bound to differ from field to field*”. We share his skepticism; perhaps it would suffice with a “reasonable” weighting matrix, the best among those considered. In practical terms, this means that the problem of selecting a weighting matrix can be interpreted as a problem of model selection. In fact, different weighting matrices result in different spatial lags of the variables included in the model and different equations with different regressors amounts to a model selection problem.

As said, our intention is to offer new evidence to help the user to select the most appropriate **W** matrix for the specification. Section 2 revises four selection criteria that fit well into the problem of selecting a weighting matrix from among a finite set of them. Section 3 presents the main features of the Monte Carlo experiment solved in the fourth section. Section 5 includes a well-known case study which is revised in the light of our findings. The sixth section concludes.

## 2. Criteria to Select a W Matrix from among A Finite Set

A general spatial panel data econometric model, can be expressed as:

(1) $$\left.\begin{array}{c} y_{t}=\rho_{1}Wy_{t}+x_{t}\beta +Wx_{t}\theta +u_{t},\\ u_{t}=\rho_{2}Mu_{t}+\varepsilon_{t}, \end{array}\right\}$$

$y_{t}$ is a (N×1) vector of data for the explained variable in period t,t=1,2,…,T; $x_{t}$ is a (N×k) matrix of observed data for the explicative variables, assumed exogenous, in period *t*; $u_{t}$ is a (N×1) vector of error terms in period *t* and $\varepsilon_{t}$ a vector of random terms, assumed to be normally distributed (this assumption can be relaxed). β, θ, $\rho_{1}$ and $\rho_{2}$ are unknown parameters; the last two parameters are called *spatial correlation* parameters. Finally, W and M are two weighting matrices, specified exogenously, that channel the corresponding spill-over effects. Usually, the two matrices are forced to be equal. The model of (1) is called a Cliff-Ord specification. A Spatial Durbin Model, SDM, results from $\rho_{2}=0$; a Spatial Lag Model, SLM, requires that θ=0 and $\rho_{2}=0$; a Spatial Durbin Error Model, SDEM, $\rho_{1}=0$ and a Spatial Error Model, SEM, that θ=0 and $\rho_{1}=0$.

In view of model (1), it is clear the critical importance played by the weighting matrices. We really need these matrices but there are few clues to build them in applied research; this results in the so-called W issue, partly review in the first section. In recent decades, a very interesting literature has appeared that examines the problem of choosing a matrix among a finite set of them, which is the target of this paper. First, we review the literature devoted to the *J* test and then we move to the selection criteria, Bayesian methods and a new procedure based on entropy.

We should recognize that there are other interesting procedures in the literature, like, for example, the model boosting approach of Kostov [23] and the model averaging of Zhang and Yu [24], which are not used in our study for reasons of space or computational burden.

Anselin [25] was the first to raise, formally, the **W** issue, suggesting a *Cox* statistic in a framework of non-nested models. Leenders [26], on this basis, elaborates a *J*-test using classical augmented regressions. Later, Kelejian [27] extends the approach of [26] to a SAC model, with spatial lags of the endogenous variable and in the error terms, using Generalized Method of Moments, GMM, estimates. Piras and Lozano [28] confirm the adequacy of the *J*-test to compare different weighting matrices stressing that we should make use of a full set of instruments to increase GMM accuracy. Burridge and Fingleton [29] show that the Chi-square asymptotic approximations for the *J*-tests produces irregular results, excessively liberal or conservative in a series of leading cases; they suggest a bootstrap resampling approach. Burridge [30] focuses on the propensity of the spatial GMM algorithm to deliver spatial parameter estimates lying outside the invertibility region which, in turn, affects the bootstrap; he suggests the use of a QML algorithm to remove the problem. Kelejian and Piras [31] extended the original version of [27] to account for all the available information, according to the findings of [28]. Finally, Kelejian and Piras [32] adapt the *J* test to a panel data setting with unobserved fixed effects and additional endogenous variables which reinforces the adequacy of the GMM framework. Another milestone in the *J* test literature is Hagemann [33], who copes with the reversion problem originated by the lack of a well-defined null hypothesis in the test. He introduces the minimum *J* test, MJ. His approach is based on the idea that if there is a finite set of competing models, only the model with the smallest *J* statistic can be the correct one. In this case, the *J* statistic will converge to the Chi-square distribution but will diverge if none of the models is correct. The author proposes a wild bootstrap to test if the model with the minimum *J* is correct. This approach has been applied by Debarsy and Ertur [20] to a spatial setting with good results.

In the Monte Carlo experiment that follows, we know that there is a correct model so are going to use only the first part of the procedure of [33]. Assuming a collection of *M* different weighting matrices, such as: $W=\left\{W_{1};W_{2};\ldots ;W_{M}\right\}$, the MJ approach works as follows:

1. We need the estimates of the *m* models; in each case, the same equation is employed but with a different weighting matrix belonging to W. Following Burridge [30] and given that our interest lies on the small sample case, the models are estimated by ML.
2. For each model, we obtain the battery of *J* statistics as usual, after estimating, also by ML, the corresponding extended equations.
3. The chosen matrix is the one associated with the minimum *J* statistic. As said, we stop the procedure here, thus avoiding the wild bootstrap to test if this matrix is, indeed, the correct one.

Another popular method for choosing between models deals with the so-called *Information Criteria*. Most are developed around a loss function, such as the *Kullback-Leibler*, KL, quantity of information which measures the closeness of two density functions. One of them corresponds to the true probability distribution that generated the data, obviously not known, the other is the distribution estimated from the data. The criteria differ in the role assigned to the aprioris and in the way of solving the approximation to the unknown true density function [34]. The two most common procedures are the AIC [35] and the Bayesian BIC criteria [36]. The first compares the models on equal basis whereas the second incorporates the notion of model of the null. Both criteria are characterized by their lack of specificity in the sense that the selected model is the closest to the true model, as measured by KL. We should note that as indicated by Potscher [37], a good global fit does not necessarily mean that the model be the best alternative to estimate the parameters of interest. AIC and BIC lead to simple expressions that depend on the accuracy of the ML estimation plus a penalty term related to the number of parameters entering the model; that is:

(2) $$\left.\begin{array}{cc} AIC(k): & -2l(\tilde{\gamma })+2k,\\ BIC(k): & -2l\left(\tilde{\gamma }\right)+k\log\left(n\right), \end{array}\right\}$$

where $l(\tilde{\gamma })$ means the estimated log-likelihood at the ML estimates, $\tilde{\gamma }$, *k* is the number of non-zero parameters in the model and *n* the number of observations. For the case that we are considering the models only differ in the weighting matrix, so *k* and *n* are the same in every case. This means that the decision depends on the estimated log-likelihood or, what is the same, on the balance between the estimated variance and the Jacobian term. Please note that for a standard SLM, we can write: $l\left(\tilde{\gamma }\right)\propto \log\left[\frac{1}{{\tilde{\sigma }}^{n}}\left|I-\tilde{\rho }W\right|\right]$, being σ the standard deviation and ρ the corresponding spatial dependence coefficient. To minimize any of the two statistics in (2) the objective is to maximize the concentrated estimated log-likelihood, $l(\tilde{\gamma })$. In sum, the *Information Criteria* approach implies:

1. Estimate by ML of the *M* models corresponding to each weighting matrix in W.
2. For each model, we obtain the corresponding AIC statistic (BIC produces the same results).
3. The matrix in the model with minimum AIC statistic should be chosen.

An important part of the recent literature on spatial econometrics has Bayesian grounds, which extends also to the topic of choosing a weighting matrix. The Bayesian framework is well equipped to cope with these types of problems using the concept of *posterior probability* as the basis for taking a decision. There are excellent reviews in [38,39,40], Besag and Higdon [41] and especially, [42,43,44]. For the sake of completeness, let us highlight the main points in this approach.

The analysis is made conditional to a model, which is not under discussion. Moreover, we have a collection of *M* weighting matrices in W, a set of *k* parameter in γ, some of which are of dispersion, σ, others of position, β, and others of spatial dependence, ρ and θ, and a panel data set with nT observations in *y*. The point of departure is the joint probability of data, parameters and matrices:

(3) $$p\left(W_{m};\gamma ;y\right)=\pi \left(W_{m}\right)\pi \left(\gamma |W_{m}\right)L\left(y|\gamma ;W_{m}\right),$$

where $W_{m}\in W$; $\pi \left(\;\cdot \;\right)$ are the prior distributions and $L\left(y|\gamma ;W_{m}\right)$ the likelihood for *y* conditional on the parameters and the matrix. Bayes’ rule leads to the posterior joint probability for matrices and parameters:

(4) $$p\left(W_{m};\gamma |y\right)=\frac{\pi \left(W_{m}\right)\pi \left(\gamma |W_{m}\right)L\left(y|\gamma ;W_{m}\right)}{L\left(y\right)},$$

whose integration over the space of parameters, γ∈Υ, produces the posterior probability for matrix $W_{m}$:

(5) $$p\left(W_{m}|y\right)=\int_{\Upsilon }p\left(W_{m};\gamma |y\right)d\gamma .$$

The presence of spatial structures in the model complicates the resolution of (5) which requires of numerical integration. The priors are always a point of concern and, usually, practitioners prefer diffuse priors. LeSage and Pace [42] (Section 6.3) suggest $\pi \left(W_{m}\right)=\frac{1}{M}\;\forall m$, a NIG conjugate prior for β and σ where $\pi_{\beta }\left(\beta |\sigma \right)∼N\left(\beta_{0};\sigma^{2}{\left(\kappa X^{\prime }X\right)}^{-1}\right)$, being *X* the matrix of the exogenous variables in the model, and $\pi \left(\sigma \right)$ an inverse gamma, IG(a,b). For the parameter of spatial dependence, they suggest a Beta(d,d) distribution, being *d* the amplitude of the sampling space of ρ. The defaults in the ${\mathrm{MATLAB}}^{®}$ codes of LeSage [45] are $\beta_{0}=0$, $\kappa =10^{-12}$ and a=b=0. In brief, the *Bayesian* approach implies the following:

1. Specify the priors for all the terms appearing in the equation. In this point, we have followed the suggestions of [42].
2. For each matrix, obtain the corresponding posterior probability of (5) for which we need (i) solve the ML estimation of the corresponding model and (ii) solve the numerical integration of (5).
3. The matrix chosen will be that associated with the highest posterior probability.

This paper advocates for a selection procedure based on the notion of *entropy*, which is used as a measure of the information contained in a distribution of probability. Let us assume a univariate continuous variable, *y*, whose probability density function is p(y); then, *entropy* is defined as:

(6) $$h\left(y\right)=-\int_{I}p\left(y\right)\log p\left(y\right)dy,$$

being *I* the domain of the random variable *y*. As known, higher *entropy* means less information and more uncertainty about *y*. Our case fits with Shannon’s framework [46]: we observe a random variable, *y*, and there is a finite set of rival distribution functions capable of having generated the data. Our decision problem is well defined because each distribution function differs from the others only in the weighting matrix; the other elements are the same. However, we are not interested in the Laplacian principle of indifference (select the density with maximum *entropy*, less informative, to avoid uncertain information). Quite the opposite: in our case there is no uncertain information and we are looking for the more informative probability distribution, so our objective is to minimize *entropy*.

As with the other three cases, the application of this principle requires the complete specification of the distribution function, which means that the user knows the form of the model (Equations (8)–(10) below, except the W matrix); additionally we add a Gaussian distribution. Next, we should remind that for the case of a (n×1) multivariate normal random variable, y∼N(μ;Σ), the entropy is: $h\left(y\right)=\frac{1}{2}\left[n+\log\left({\left(2\pi \right)}^{n}\left|\Sigma \right|\right)\right]$. This measure does not depend, directly, on first order moments (parameters of position of the model) but on second order moments (dependence and dispersion parameters). For example, in the case of the SLM of (10) below, the entropy is:

(7) $$h{\left(y\right)}_{SDM}=\frac{1}{2}{\left(nT+\log((2\pi \sigma^{2}\right)}^{nT}\left|{\left(B^{\prime }B\right)}^{-1}\right|))$$

where $B=\left(I-\rho W\right)$. Please note that the covariance matrix for *y* in the SDM is $V\left(y\right)=B^{-1}V\left(u\right)B^{\prime -1}$. If *u* is indeed a white noise random term with variance $\sigma^{2}$, the covariance matrix of *y* is simply $V\left(y\right)=\sigma^{2}{\left(B^{\prime }B\right)}^{-1}$. Let us note that the covariance matrix of *y* in the SDM of (8) coincides with that of the SLM case. The covariance matrix for the SDEM equation is $V\left(y\right)=\sigma^{2}\left(B^{\prime }B\right)$, everything else remains the same.

To apply the *Entropy* criterion we must go through the following steps:

1. Estimate each one of the *M* versions of the model that we are considering. As said, each model differs only in the weighting matrix. We obtain the ML estimates for reasons given above.
2. For each model, we obtain the corresponding value of the *entropy*, in the $h_{m};\;m=1,2,\ldots ,M$ statistic.
3. The decision criterion consists in choosing the weighting matrix corresponding to the model with minimum value of *entropy*.

## 3. Description of the Monte Carlo Study

This part of the paper is devoted to the design of the Monte Carlo experiment conducted to calibrate the performance of the four criteria presented so far for selecting W: the MJ procedure, the *Bayesian* approach, the AIC criterion and the *entropy* measure. The objective of the analysis is to identify the most reliable method to select the most adequate weighting matrix for a spatial model, given the data of the variables and the form of the model itself. The parameters are also unknown for the user and must be estimated. In this context, if the matrix is misspecified, and the estimated parameters will be biased, which will impact the four criteria described in Section 2 in different ways. Our Monte Carlo study generates sequences of data of the explained and explicative variables, for different scenarios, and applies the four criteria to select the (unknown) **W**. Moreover, our focus is on small sample sizes. As will be clear soon, the four criteria have good behavior even in small samples, so it is not necessary to employ very large sample sizes.

We are going to simulate a panel setting, with three of the most common Data Generating Processes, *DGPs* in what follows, in the applied literature on spatial econometrics; namely, the spatial Durbin Model, SDM of (8), the Spatial Durbin Error Model, SDEM in expression (9) and the Spatial Lag Model of (10), SLM. Main conclusions can be extended to other processes such as the Spatial Error Model, which are not replicated here (details on request from the authors).

(8) $$\begin{array}{cc} y_{it} & =\beta_{0}+\rho \sum_{j=1}^{n}\omega_{ij}y_{jt}+x_{it}\beta_{1}+\theta \sum_{j=1}^{n}\omega_{ij}x_{jt}+\varepsilon_{it}, \end{array}$$

(9) $$\begin{array}{cc} y_{it} & =\beta_{0}+x_{it}\beta_{1}+\theta \sum_{j=1}^{n}\omega_{ij}x_{jt}+u_{it},\;u_{it}=\rho \sum_{j=1}^{n}\omega_{ij}u_{jt}+\varepsilon_{it}. \end{array}$$

(10) $$\begin{array}{cc} y_{it} & =\beta_{0}+\rho \sum_{j=1}^{n}\omega_{ij}y_{jt}+x_{it}\beta_{1}+\varepsilon_{it}, \end{array}$$

Only one exogenous regressor, *x* variable, appears in the right hand side of the equations whose observations are obtained from a normal distribution, $x_{it}∼i.i.d.N\left(0;\sigma_{x}^{2}\right)$, where $\sigma_{x}^{2}=1$; the same applies with respect to the error terms: $\varepsilon_{it}∼i.i.d.N\left(0;\sigma_{\varepsilon }^{2}\right)$, where $\sigma_{\varepsilon }^{2}=1$. The two variables are not related, $E\left(x_{it}\varepsilon_{it}\right)=0$. Our space is made of hexagonal pieces which are arranged regularly, one next to the others without discontinuities nor empty spaces.

A weighting matrix appears in the three equations, which is not observable, and the user must take decisions to continue with the analysis. The problem consists in choosing one matrix from among a finite set of alternatives which in our simulation are composed by three candidates: $W_{1}$ is built using the traditional contiguity criterion between spatial units; the weights in $W_{2}$ are the inverse of the distance between the centroids of the spatial units, $W_{2}=\left\{\omega_{ij}=\frac{1}{d_{ij}};i\neq j\right\}$; whereas $W_{3}$ incorporates a cut-off point to the connections in $W_{2}$, so that $W_{3}=\left\{\omega_{ij}=\frac{1}{d_{ij}};i\neq j\;if\;j\in N_{4}\left(i\right);\;0\;otherwise\right\}$ being $N_{4}\left(i\right)$ the set of 4 nearest neighbors to *i*. To keep things simple, the same weighting matrix appears with the endogenous and exogenous variables in (8) and with the exogenous and error terms in (9). Following usual practice, every matrix has been row-standardized, which implies that the three matrices are non-nested. In what follows we will use $W_{3}$ as the true matrix.

Three different small cross-sectional sample sizes, *n*, have been used $n\in \left\{25,49,100\right\}$; that is enough because higher values of this parameter only improve marginally the results. For the same reason, the number of cross-sections in the panel, *T*, are limited to only three, $T\in \left\{1,5,10\right\}$. The values for the coefficient of spatial dependence, ρ, ranges from negatives to positives, $\rho =\left\{-0.8,-0.5,-0.2,0.2,0.5,0.8\right\}$. Other global parameters are those associated with the constant term, $\beta_{0}=1$, the *x* variable, $\beta_{1}\in \left\{1,5\right\}$, and its spatial lag, $\theta \in \left\{1,5\right\}$.

In sum, each case consists in:

- Generate the data using a given weighting matrix, $W_{m},\;m=1,2,3$ and a spatial equation, SLM, SDM, or SDEM. There are 216 cases of interest for each equation (6 values in ρ, 3 values in *n*, 3 values in *T*, 2 values in $\beta_{1}$ and 2 values in θ).
- The spatial equation is assumed to be known so the model can be estimated by maximum likelihood, ML, once the user supplies a W matrix.
- Compute the four selection criteria, MJ, *Posterior probability*, *entropy* and AIC for the three alternative weighting matrices for each draw.
- Select the corresponding matrix according to each criterion and compare the result with the *true* matrix ($W_{3}$) in the DGP.
- The process has been replicated 1000 times.

Please note that the selection of the matrix is made conditional on a correct specification of the equation. We are perfectly aware that this dichotomy is artificial; in fact, both decisions are intimately related because the same matrix, but in different equations, plays different roles and bears different information. However, this point is not further developed in the present paper. To give some intuition, we include the results corresponding to the case of a wrong specification (i.e., estimate a SDM model whereas the true model in the DGP is a SDEM). MATLAB^®^ codes to replicate these simulations are freely downloadable from https://sites.google.com/site/mherreragomez/principal/Codes.

## 4. Results of the Monte Carlo Study

This section summarizes the results obtained in the Monte Carlo simulation described previously and, we must admit, they are a bit surprising: in strictly quantitative terms, the AIC and the *entropy* measures are the best criteria. What is more striking, according to our results the *Bayesian* approach, although it does well in general, it is clearly the third criterion. Finally, the MJ approach is the worst alternative among the four candidates. The last two observations are puzzling given the strong support that the two procedures have received in recent decades. Table 1 presents the percentage of correct selections attained by each criterion after aggregating all the cases in our simulation. Each percentage accumulates 126,000 items. A number in **bold** indicates that the respective criterion reaches the maximum rate of correct selections.

Entropy dominates at the extremes of the range of values for the spatial dependence coefficient, whereas AIC is the best for medium to low values of ρ. The differences between the two are always lower to 3.5 percentage points (in fact, the average proportion of correct selections is statistically equal with a confidence of 99%). Bayes is a good criterion for medium to large values of ρ but its performance weakens for small values of this parameter (in fact, is fourth in ±0.2). Finally, the curve of correct selections of the MJ is too flat.

**Figure 1:**
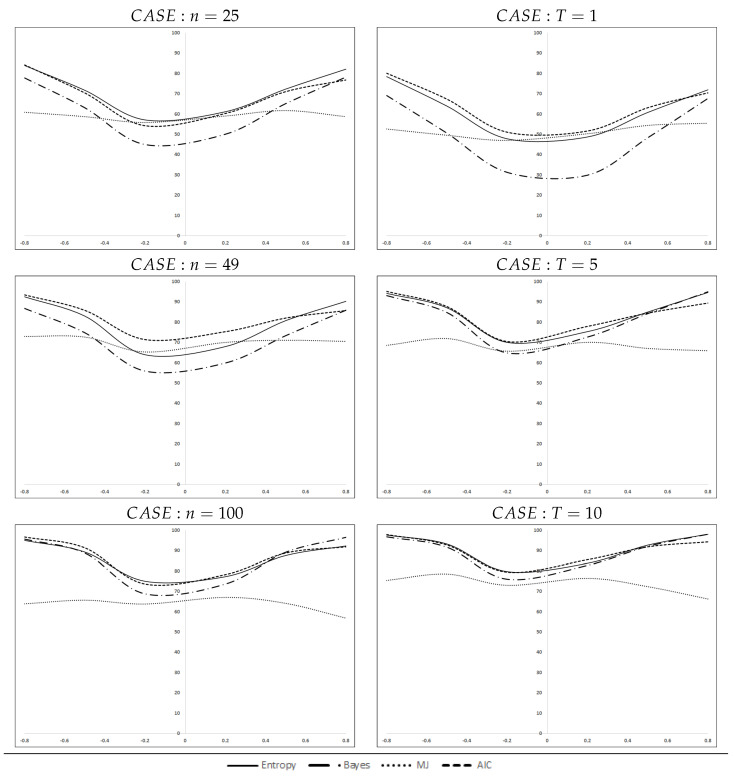
Percentages of correct selections, disaggregated by *n* and *T*.

Figure 1 disaggregates the accumulated percentages by number of spatial units, left, or number of cross-sections, right. Please note that in each case, the data represent aggregated percentages (i.e., in the case n=25 we aggregate the three cross-sections corresponding to T=1, T=5 and T=10). These figures ratify the ordering set out above. The behavior of the MJ criterion is striking: its curves of correct selections are very flat, with unexpected drops at the extremes of the interval for ρ when the sample size (*n* or *T*) increases. The other three criteria, as expected, react positively to the sample size or to higher values of ρ. Apparently, the improvement is more relevant for the time dimension, *T*, than for the cross-sectional size *n*, especially for high values of the spatial coefficient. Finally, there is a certain asymmetry in all the curves.

Table 2, Table 3, Table 4 and Table 5 present the details by type of DGP. A quick look at the tables reveals that bold percentages are concentrated, mainly, in the *entropy* and AIC columns.

The prevalence of both criteria is quite regular for the four cases shown in the tables. The preference extends to the case of correctly specified models, as in Table 2, Table 3 and Table 4, and also for misspecified equations, as in Table 5, for negative and especially for positive values of the spatial coefficient, for small and large number of individuals in the sample (*n*) and for simple to large panels (*T*). Overall, *entropy* attains the highest rate in 46% of the 144 cases in Table 2, Table 3, Table 4 and Table 5, followed by AIC, 35%, *Bayes*, 12%, and MJ, 7%.

The complete relation of results for the 756 different experiments in the study of Monte Carlo (3 *n*s, 3 *T*s, 6 ρs, 2 βs, 2 θs and four configurations for the DGP/estimated equation pair; note that the parameter θ does not intervene in the SLM equation) appear in Table A1–Table A12 in the Appendix. We want to stress the good results attained in the case of small samples (n=25 and T=1) where the average rate of correct selections for *entropy* and AIC is usually above 30% (a little worse for the other two criteria). Very often, the percentage exceeds 70% at the extremes of the spatial parameter interval, ±0.8. The average rate increases up to 75–80%, for the case of n=25 and T=5 and continues improving when T=10, where most cases have a rate of correct selections above 90%. In general, the rate of correct selections is nearly 100%, using 5 to 10 cross-sections.

In a similar vein, the increase in the cross-sectional size, *n*, when the number of cross-sections, *T*, remains constant also has positive effects in the four criteria. The rate of correct selections for the case of a hundred of spatial units is above 70%, on average, for the case of a single cross-section (T=1). These percentages improve quickly once the time dimension of the panel increases; it is also clear that the improvement depends on the type of DGP (stronger for SDM or SLM models and weaker for the SDEM and for the misspecified equation case).

The value of parameter $\beta_{1}$, as expected, has only a slight impact in the four criteria; on the contrary, the signal of $\theta_{1}$ plays a crucial role in the SDEM and SDM cases. Another interesting feature is the asymmetry of the selection curves that tends to be diluted with *T*. Negative spatial dependence helps to better detect the correct weighting matrix, especially when the number of time cross-sections is small. The asymmetry is evident in *entropy*, *Bayes* and AIC, but it is more diffused in the MJ case which remains highly inelastic to the value of ρ.

To complete the picture, we estimate a *response-surface* for each DGP/Estimated equation combination, with the aim of modelling the empirical probability of choosing the correct weighting matrix for each criterion, $p_{i}$. As usual, a logit transformation of the empirical probabilities is carried out, so the estimated equation is:

(11) $$\log\left(\frac{p_{i}+{\left(2r\right)}^{-1}}{1-p_{i}+{\left(2r\right)}^{-1}}\right)=p_{i}^{*}=\eta +z_{i}\varphi +\epsilon_{i},$$

where $p_{i}^{*}$ is the logit transformation, often known as the *logit*, *r* the number of replications of each experiment (1000 in all the cases); ${\left(2r\right)}^{-1}$ assures that the *logit* is defined even when the probability of correct selection is 0 or 1 [47]; η is an intercept term, $z_{i}$ the design matrix whose columns reflect the conditions of each experiment, φ is a vector of parameters and $\epsilon_{i}$ the error term assumed to be independent and identically distributed (this assumption is reasonable if all experiments come from the same study, as ours, and are obtained under identical circumstances; [48]). Recall that the number of observations in the *response-surface* equations is 216 (so i=1,2,…,216), except for the SLM case where the number of observations is 108. Table 6 shows the results for the four DGP/Estimated equation combinations.

In general, the estimates confirm previous facts. The main factor influencing the empirical probability of choosing the correct weights matrix is the spatial parameter, absolute value of ρ in Table 6. Its contribution is crucial in all the cases, without exceptions, and occurs in the expected direction: higher values of $\left|\rho \right|$ facilitate the selection of the correct weighting matrix. The second more influential factor is the parameter θ, associated with spatial spillovers. Also, its impact is beneficial for all the cases though it appears to be more important for the *Bayesian* and MJ criteria. Sample size is crucial and *T* has a relatively higher impact than *n*. Finally, as said before, parameter $\beta_{1}$ is not significant in any circumstance, except for the SLM case; this means that the *signal-to-noise* ratio should not be a major factor to consider when the problem is selecting the best weighting matrix.

Table 7 completes the *response-surface* analysis with the *F* tests of equality in the coefficients of the estimates of Table 6. According to the sequence of *F* tests, the most dissimilar method is the MJ approach, and then *Bayes*. On the other hand, *entropy* and AIC emerge as quasi-similar strategies to compare weighting matrices, almost indistinguishable in the four types of DGPs.

## 5. Empirical Application: Ertur and Koch (2007)

The case study in this section is based on a well-known economic growth model estimated by Ertur and Koch (2007) using a cross-section of 91 countries for the period 1960–1995. The purpose of this section is to illustrate the use of the selection procedures discussed before.

Ertur and Koch [49] build a growth equation to model technological interdependence between countries using spatial externalities. The main hypotheses of interaction are that the stock of knowledge in one country produces externalities that cross-national borders and spill over into neighboring countries, with an intensity which decreases with distance. The authors use a geographical distance measure.

The benchmark model assumes an aggregated Cobb-Douglas production function with constant returns to scale in labor and physical capital:

(12) $$Y_{i}\left(t\right)=A_{i}\left(t\right)K_{i}^{\alpha }\left(t\right)L_{i}^{1-\alpha }\left(t\right),$$

where $Y_{i}\left(t\right)$ is output, $K_{i}\left(t\right)$ is the level of reproducible physical capital, $L_{i}\left(t\right)$ is the level of labor in the period *t*, and $A_{i}\left(t\right)$ is the aggregate level of technology specified as:

(13) $$A_{i}\left(t\right)=\Omega \left(t\right)k_{i}^{\phi }\left(t\right)\prod_{j\neq i}^{n}A_{i}^{\delta \omega_{ij}}\left(t\right).$$

The aggregate level of technology $A_{i}\left(t\right)$ in a country *i* depends on three elements. First, a certain proportion of technological progress is exogenous and identical in all countries: $\Omega \left(t\right)=\Omega \left(0\right)e^{\mu t}$, where μ is a constant rate of technological growth. Second, each country’s aggregate level of technology increases with the aggregate level of physical capital per worker $k_{i}^{\phi }\left(t\right)={\left(K_{i}\left(t\right)/L_{i}\left(t\right)\right)}^{\phi }$ with parameter $\phi \in \left[0;1\right]$ capturing the strength of home externalities by physical capital accumulation. Finally, the third term captures the external effects of knowledge embodied in capital located in a different country, whose impact crosses national borders at a diminishing intensity, $\delta \in \left[0;1\right]$. The terms $\omega_{ij}$ represent the connectivity between country *i* and its neighbors; these weights are assumed to be exogenous, non-negative, and finite.

Following Solow, the authors assume that a constant fraction of output $s_{i}$, in every country *i*, is saved and that labor grows exogenously at the rate $l_{i}$. Also, they assume a constant and identical annual rate of depreciation of physical capital for all countries, denoted τ (assumed as a constant value equal 0.05 in the literature). The evolution of output per worker in country *i* is governed by the usual fundamental dynamics of the Solow equation which, after some manipulations, lead to a steady-state real income per worker [49] (p. 1038, Equation (9)):

(14) $$y=\Omega +\left(\alpha +\phi \right)k-\alpha \delta Wk+\delta Wy.$$

This is a spatially augmented Solow model and coincides with the predictor obtained by Solow adding spill-over effects. In terms of spatial econometrics, we have a *Spatial Durbin Model*, SDM, which can be expressed as:

(15) y=xβ+ρWy+Wxθ+ε.

Equation (15) is estimated using information on real income, investment and population growth for a sample of 91 countries for the period 1960–1995. Regarding the spatial weighting matrix, [49] consider two geographical distance functions: the inverse of squared distance (which is the main hypothesis) and the negative exponential of squared distance (to check robustness in the specification). We also consider a third matrix based on the inverse of the distance.

Let us call the three weighting matrices as $W_{1}$, $W_{2}$ and $W_{3}$ which are row-standardized: $\omega_{hij}=\omega_{hij}^{*}/\sum_{j=1}^{n}\omega_{hij}^{*};\;h=1,2,3$ where:

(16) $$\begin{array}{ccc} \omega_{1ij}^{*}=\left\{\begin{array}{cc} 0 & if\;i=j\\ d_{ij}^{-2} & otherwise \end{array};\right. & \omega_{2ij}^{*}=\left\{\begin{array}{cc} 0 & if\;i=j\\ e^{-2d_{ij}} & otherwise \end{array};\right. & \omega_{3ij}^{*}=\left\{\begin{array}{cc} 0 & if\;i=j\\ d_{ij}^{-1} & otherwise \end{array}\right., \end{array}$$

with $d_{ij}$ as the great-distance (i.e., the shortest distance between two points on the surface of a sphere) between the capitals of countries *i* and *j*.

The authors analyze several specifications checking for different theoretical restrictions and alternative spatial equations. We concentrate our revision in the so-called non-restricted equation, in the sense that it includes more coefficients than advised by theory. Table 8 presents the SDM version of this equation using the three alternative weighting matrices specified before (the first two columns coincide with those in Table I, columns 3–4, pp. 1047, of [49]). The last four rows in the Table show the value of the selection criteria corresponding to each case.

The preferred model by [49] is the $SDM/W_{1}$ which coincides with the selection attained by minimum *entropy*, the *Bayesian* posterior probability and AIC. The selection of the MJ approach is $W_{2}$.

Other results in [49] refer to the Spatial Error Model version of the steady-state equation of (14), or SEM model. The intention of the authors is to visualize the presence of spatial correlation in the traditional non-spatial Solow equations; we use this case as an example of selection of weighting matrices in misspecified models. The main results appear in Table 9 (which can be compared with columns 2–3 of Table II, in [49] (p. 1048)).

The selection of the most adequate W matrix does not change. Using the values of *entropy* criterion we select the model in which intervenes the matrix $W_{1}$, the same as with the Bayesian approach and the AIC criterion; MJ continues selecting $W_{2}$.

## 6. Conclusions

Much of the applied spatial econometrics literature seems to prefer an exogenous approximation to the W matrix. Implicitly, it is assumed that the user has relevant knowledge with respect to the way individuals in the sample interact. In recent years, new literature advocates for a more data driven approach to the W issue. We strongly support this tendency, which should be dominant in the future; however, our focus in this study is on the exogenous approach.

The problem posed in the paper is very frequent in applied work: the user has a finite collection of weighting matrices, they all are coherent with the case of study, and one needs to select one of them. Which is the best W? We can address this question using different proposals: the *Bayesian* posterior probability, the *J* approach with all its variants, by means of simple model selection criteria, such as AIC or BIC and several other alternatives not used in this study. We add a fourth one, based on the *entropy* of the estimated distribution function. This new criterion *h(y)* is a measure of uncertainty, and fits well with the W decision problem. The *h(y)* statistics depends on the estimated covariance matrix of the corresponding model offering a more complete picture of the suitability of the distribution function (related to a particular choice of W), to deal with the data at hand.

The conclusions of our Monte Carlo experiment are very illuminating. First, we can confirm that it is possible to identify, with confidence, the true weighting matrix (if it really exists); in this sense, the selection criteria do a good job. However, the four criteria should not be taken as indifferent, especially in samples of small size (*n* or *T*). The ordering is clear: *entropy* and AIC in first place and then *Bayesian* posterior probability doing slightly worse; the MJ appears always in the last position. As shown in the paper, the value of the spatial parameter has a great impact to guarantee a correct selection, but this aspect is unobservable to the researcher. However, the user effectively controls the amount of information involved in the exercise, and this is also a key factor. The advice is clear: use as much information as you have because the quality of the decision improves with the amount of information. Once again, the way the information accrues is not neutral: the length of the time series in the panel is more relevant than the number of cross-sectional units in the sample.

Our final recommendation for applied researchers is to care for the adequacy of the weighting matrix and, in case of having various candidates, take a decision using well-defined criteria such as those examined in the paper. The case of study presented in Section 5 illustrates the procedure.

As avenues for future research, let us mention the possibility of combining different matrices into a single one, as pursued in model averaging or in fuzzy logic, which offers new, flexible alternatives. Moreover, this study assumes that the user knows the form of the equation which is, very often, an unrealistic assumption. This constraint poses new challenges and can be solved by using a more general framework where both the model and the matrix should be chosen. It is clear that not all the four criteria are well equipped to work in the new scenarios.

## Figures and Tables

**Table 1:** Percentage of correct selections. Aggregated results.

ρ	h(y)	*Bayes*	MJ	AIC
−0.8	**91.52**	86.81	65.91	91.50
−0.5	81.37	75.62	65.71	**82.58**
−0.2	65.38	56.59	61.69	**66.44**
0.2	68.82	61.25	65.40	**71.31**
0.5	80.25	75.97	65.67	**80.68**
0.8	**88.27**	86.95	62.04	84.83
AVERAGE	79.27	73.86	64.40	**79.56**

**Table 2:** Average percentage of correct selections. DGP: SDM. Equation estimated: SDM.

Aggregated by Cross-Section, Sample Size (*n*)						Aggregated by Time Series, Sample Size (*T*)					
	ρ	h(y)	*Bayes*	MJ	AIC		ρ	h(y)	*Bayes*	MJ	AIC
n=25	−0.8	**90.40**	84.32	68.28	90.36	T=1	−0.8	**87.84**	80.77	68.46	87.33
	−0.5	**79.08**	67.93	65.73	78.85		−0.5	**73.13**	53.77	62.86	71.98
	−0.2	62.18	44.87	**62.68**	59.53		−0.2	56.88	29.17	**60.19**	54.78
	0.2	**74.18**	58.46	74.13	73.74		0.2	64.84	33.33	**66.28**	63.67
	0.5	84.46	71.64	77.42	**84.91**		0.5	79.90	58.01	69.93	**81.14**
	0.8	91.81	85.32	78.24	**93.11**		0.8	88.94	81.32	70.91	**91.20**
n=49	−0.8	**96.01**	94.85	78.65	95.70	T=5	−0.8	99.38	**99.46**	79.33	99.30
	−0.5	**90.50**	83.31	81.11	89.71		−0.5	**94.38**	93.56	78.13	94.05
	−0.2	**75.32**	61.16	72.32	73.45		−0.2	**76.32**	67.28	71.34	74.22
	0.2	**84.07**	72.08	82.32	83.95		0.2	88.48	85.89	86.36	**88.97**
	0.5	89.83	80.58	76.65	**90.43**		0.5	96.16	96.20	89.93	**96.72**
	0.8	96.97	95.98	87.03	**97.87**		0.8	99.09	99.13	89.76	**99.38**
n=100	−0.8	**99.29**	99.28	85.22	99.15	T=10	−0.8	98.46	98.22	84.35	**98.58**
	−0.5	**96.74**	94.76	88.09	96.17		−0.5	**98.81**	98.67	93.94	98.69
	−0.2	**84.68**	75.48	78.26	83.59		−0.2	**88.98**	85.07	81.72	87.57
	0.2	91.76	84.64	89.62	**91.89**		0.2	96.68	95.97	93.42	**96.95**
	0.5	96.09	94.14	84.30	**96.63**		0.5	**94.33**	92.16	78.52	94.12
	0.8	99.08	99.01	92.22	**99.52**		0.8	99.83	99.86	96.82	**99.91**

**Table 3:** Average percentage of correct selections. DGP: SDEM. Equation estimated: SDEM.

Aggregated by Cross-Section, Sample Size (*n*)						Aggregated by Time Series, Sample Size (*T*)					
	ρ	h(y)	*Bayes*	MJ	AIC		ρ	h(y)	*Bayes*	MJ	AIC
n=25	−0.8	80.33	79.19	57.23	**80.91**	T=1	−0.8	74.32	71.68	46.25	**74.80**
	−0.5	**68.56**	66.58	56.45	67.95		−0.5	63.12	60.62	46.43	**65.18**
	−0.2	**59.53**	54.35	57.35	57.24		−0.2	49.71	44.53	45.89	**49.98**
	0.2	**56.63**	51.63	55.81	55.34		0.2	45.94	36.60	**47.39**	43.81
	0.5	**66.25**	63.64	56.66	64.05		0.5	**54.96**	48.89	49.19	52.76
	0.8	**77.18**	75.99	54.48	68.94		0.8	**65.64**	64.66	51.28	58.78
n=49	−0.8	91.19	90.30	67.18	**91.93**	T=5	−0.8	94.32	94.76	71.17	**95.35**
	−0.5	81.77	80.88	67.14	**82.93**		−0.5	84.92	85.15	71.55	**85.44**
	−0.2	**68.18**	63.65	67.10	66.62		−0.2	**72.32**	67.68	72.23	69.79
	0.2	**67.90**	62.95	66.42	66.66		0.2	**72.97**	68.88	70.24	72.23
	0.5	**78.96**	76.93	65.26	76.23		0.5	84.98	**85.15**	67.63	82.88
	0.8	87.78	**87.93**	62.95	79.50		0.8	94.68	**94.83**	64.07	85.83
n=100	−0.8	95.14	95.05	71.12	**95.69**	T=10	−0.8	98.03	98.10	78.11	**98.38**
	−0.5	89.04	89.13	72.64	**90.53**		−0.5	**91.33**	90.82	78.24	90.79
	−0.2	**75.08**	70.90	72.44	73.80		−0.2	**80.76**	76.69	78.78	77.89
	0.2	**75.79**	70.69	71.91	74.96		0.2	**81.41**	79.79	76.50	80.93
	0.5	**88.23**	87.20	70.23	86.56		0.5	93.50	**93.73**	75.33	91.21
	0.8	94.44	**94.72**	67.50	89.46		0.8	99.08	**99.15**	69.58	93.29

**Table 4:** Average percentage of correct selections. DGP: SLM. Equation estimated: SLM.

Aggregated by Cross-Section, Sample Size (*n*)						Aggregated by Time Series, Sample Size (*T*)					
	ρ	h(y)	*Bayes*	MJ	AIC		ρ	h(y)	*Bayes*	MJ	AIC
n=25	−0.8	92.07	**92.43**	74.40	91.87	T=1	−0.8	**89.88**	89.30	67.97	89.03
	−0.5	**77.87**	77.35	60.05	77.50		−0.5	**69.30**	58.70	53.77	67.97
	−0.2	**43.62**	36.25	39.27	41.48		−0.2	34.02	10.78	**38.20**	33.15
	0.2	54.87	44.84	39.96	**55.07**		0.2	**42.77**	10.72	40.27	42.17
	0.5	79.00	76.67	53.85	**80.23**		0.5	70.63	54.45	51.55	**73.13**
	0.8	89.25	89.05	60.03	**89.80**		0.8	84.33	82.18	59.18	**86.90**
n=49	−0.8	**97.55**	96.80	78.28	96.93	T=5	−0.8	99.48	**99.65**	84.55	99.38
	−0.5	**87.87**	81.28	67.02	87.07		−0.5	93.65	**93.95**	71.55	93.12
	−0.2	**53.60**	34.68	48.25	50.17		−0.2	**60.38**	44.75	52.08	56.87
	0.2	60.25	41.60	47.08	**61.28**		0.2	68.10	55.50	46.28	**69.88**
	0.5	86.40	76.72	61.67	**88.70**		0.5	91.55	91.85	62.23	**93.55**
	0.8	94.68	92.98	66.73	**96.47**		0.8	98.00	98.05	67.20	**98.52**
n=100	−0.8	**99.67**	99.65	87.55	99.52	T=10	−0.8	99.92	**99.93**	87.72	99.90
	−0.5	**95.45**	92.50	76.88	94.52		−0.5	98.23	**98.48**	78.63	98.00
	−0.2	**70.98**	51.47	57.78	69.10		−0.2	**73.80**	66.87	55.02	70.73
	0.2	74.12	54.75	52.66	**75.32**		0.2	78.38	74.98	53.15	**79.62**
	0.5	93.45	89.95	68.63	**95.08**		0.5	96.67	97.03	70.37	**97.33**
	0.8	97.98	97.77	71.70	**98.87**		0.8	99.58	99.57	72.08	**99.72**

**Table 5:** Average percentage of correct selections. DGP: SDEM. Equation estimated: SDM.

Aggregated by Cross-Section, Sample Size (*n*)						Aggregated by Time Series, Sample Size (*T*)					
	ρ	h(y)	*Bayes*	MJ	AIC		ρ	h(y)	*Bayes*	MJ	AIC
n=25	−0.8	77.12	62.97	50.41	**77.46**	T=1	−0.8	71.73	49.09	44.37	**74.00**
	−0.5	**64.53**	47.93	53.44	60.80		−0.5	60.23	32.04	45.41	**62.12**
	−0.2	**56.47**	40.20	55.94	52.30		−0.2	**48.33**	19.76	48.22	47.94
	0.2	55.91	42.99	**56.79**	54.41		0.2	45.56	16.46	**50.05**	43.22
	0.5	**62.99**	54.76	55.11	59.56		0.5	**52.08**	31.15	50.93	48.84
	0.8	**73.98**	68.31	42.84	61.81		0.8	**60.96**	53.97	46.86	53.86
n=49	−0.8	88.31	79.64	60.95	**89.76**	T=5	−0.8	91.93	89.32	63.69	**92.90**
	−0.5	77.69	64.33	62.45	**78.06**		−0.5	**80.93**	73.92	65.91	80.01
	−0.2	63.09	46.98	**64.23**	60.24		−0.2	66.81	56.01	**68.20**	62.62
	0.2	65.28	51.30	**65.67**	63.33		0.2	**70.52**	63.42	68.43	69.30
	0.5	**75.63**	68.70	62.52	70.78		0.5	82.70	**83.06**	64.04	78.24
	0.8	**84.84**	83.62	48.60	72.95		0.8	93.18	**93.96**	45.78	79.33
n=100	−0.8	94.48	90.80	67.81	**95.63**	T=10	−0.8	**96.24**	95.00	71.11	95.94
	−0.5	88.48	79.73	69.66	**89.80**		−0.5	**89.53**	86.03	74.23	86.54
	−0.2	**73.88**	59.86	72.84	72.03		−0.2	**78.29**	71.27	76.59	74.02
	0.2	**76.56**	64.72	73.28	75.44		0.2	**81.68**	79.13	77.26	80.66
	0.5	**88.05**	83.40	69.83	84.76		0.5	91.89	**92.65**	72.48	88.02
	0.8	93.79	**94.76**	38.97	85.73		0.8	98.48	**98.76**	37.78	87.29

**Table 6:** Estimated response surfaces.

***SDEM case***	constant	*n*	*T*	$\beta_{1}$	θ	$\left|\rho \right|$	$R^{2}$	$F_{AV}$
Entropy	−4.9179	0.0308	0.4218	−0.0103	0.6617	2.9688	0.81	183.15
	(0.0000)	(0.0000)	(0.0000)	(0.7924)	(0.0000)	(0.0000)		(0.0000)
Bayes	−5.376	0.032	0.442	−0.0107	0.6783	3.3122	0.81	184.23
	(0.0000)	(0.0000)	(0.0000)	(0.7921)	(0.0000)	(0.0000)		(0.0000)
MJtest	−5.2726	0.0309	0.4322	−0.0089	0.7447	2.5502	0.81	185.08
	(0.0000)	(0.0000)	(0.0000)	(0.8267)	(0.0000)	(0.0000)		(0.0000)
AIC	−5.1540	0.0316	0.4311	−0.0079	0.6669	3.0933	0.80	176.77
	(0.0000)	(0.0000)	(0.0000)	(0.5407)	(0.0000)	(0.0000)		(0.0000)
***SDM case***	constant	***n***	*T*	$\beta_{1}$	θ	$\left|\rho \right|$	$R^{2}$	$F_{AV}$
Entropy	−3.6764	0.0338	0.4569	0.0585	0.5613	3.0354	0.69	98.47
	(0.0000)	(0.0000)	(0.0000)	(0.2814)	(0.0000)	(0.0000)		(0.0000)
Bayes	−5.5876	0.0384	0.5720	0.0530	0.6044	4.0518	0.71	106.48
	(0.0000)	(0.0000)	(0.0000)	(0.3984)	(0.0000)	(0.0000)		(0.0000)
MJtest	−4.5043	0.0353	0.5130	0.0680	0.5880	3.0846	0.69	95.05
	(0.0000)	(0.0004)	(0.0000)	(0.0813)	(0.0000)	(0.0000)		(0.0000)
AIC	−4.3524	0.0351	0.5113	0.0494	0.5509	3.3920	0.69	97.12
	(0.0000)	(0.0000)	(0.0000)	(0.4001)	(0.0000)	(0.0000)		(0.0000)
***SLM case***	constant	***n***	*T*	$\beta_{1}$		$\left|\rho \right|$	$R^{2}$	$F_{AV}$
Entropy	−5.3523	0.0297	0.3636	0.2549		7.5807	0.86	159.69
	(0.0000)	(0.0000)	(0.0000)	(0.0000)		(0.0000)		(0.0000)
Bayes	−6.8012	0.0266	0.4475	0.2748		9.1257	0.87	180.30
	(0.0000)	(0.0000)	(0.0000)	(0.0000)		(0.0000)		(0.0000)
MJtest	−6.2131	0.0277	0.3779	0.3469		8.0125	0.83	126.07
	(0.0000)	(0.0000)	(0.0006)	(0.0000)		(0.0004)		(0.0000)
AIC	−5.8124	0.0278	0.3909	0.2495		8.2025	0.85	154.59
	(0.0000)	(0.0000)	(0.0000)	(0.0003)		(0.0000)		(0.0000)
***MISSPE case***	constant	***n***	*T*	$\beta_{1}$	θ	$\left|\rho \right|$	$R^{2}$	$F_{AV}$
Entropy	−4.5467	0.0328	0.3768	0.0001	0.4651	2.6832	0.78	153.03
	(0.0000)	(0.0000)	(0.0000)	(0.9591)	(0.0000)	(0.0000)		(0.0000)
Bayes	−6.6864	0.0372	0.5024	0.0070	0.5351	3.6498	0.82	203.30
	(0.0000)	(0.0000)	(0.0000)	(0.8627)	(0.0000)	(0.0000)		(0.0000)
MJtest	−4.9274	0.0325	0.4019	0.0079	0.5097	2.0232	0.75	131.92
	(0.0000)	(0.0000)	(0.0000)	(0.8494)	(0.0000)	(0.0000)		(0.0000)
AIC	−5.2308	0.0346	0.4190	0.0071	0.4671	2.8932	0.78	152.08
	(0.0000)	(0.0000)	(0.0000)	(0.8593)	(0.0000)	(0.0000)		(0.0000)

Note: *p*-value appears in parenthesis. $F_{AV}$ means *F* test of the null that all coefficients are zero except the constant. MISSPE means that the model in the DGP is a SDEM but we estimate an SDM equation.

**Table 7:** F test for the equality of coefficients in the response-surface estimates.

***SDEM case***	Bayes	MJtest	AIC
Entropy	0.551 (0.773)	2.593 (0.019)	0.161 (0.988)
Bayes	–	2.043 (0.062)	0.551 (0.773)
MJtest	–	–	1.993 (0.069)
***SDM case***	Bayes	MJtest	AIC
Entropy	4.652 (0.000)	1.682 (0.126)	1.066 (0.386)
Bayes	–	1.467 (0.193)	1.762 (0.109)
MJtest	–	–	0.312 (0.934)
***SLM case***	Bayes	MJtest	AIC
Entropy	8.093 (0.001)	3.124 (0.011)	0.913 (0.476)
Bayes	–	2.386 (0.043)	2.544 (0.033)
MJtest	–	–	1.743 (0.132)
***MISS case***	Bayes	MJtest	AIC
Entropy	14.085 (0.000)	5.743 (0.000)	2.086 (0.056)
Bayes	–	7.901 (0.000)	5.907 (0.000)
MJtest	–	–	2.592 (0.019)

Note: *p*-value appears in parenthesis.

**Table 8:** Ertur & Koch case. Unrestricted SDM estimates.

*Model/Weight Matrix*	*SDM / W1*	*SDM / W2*	*SDM / W3*
constant	0.967(0.610)	0.499(0.787)	5.197(0.322)
log(s)	0.825(0.000)	0.792(0.000)	0.910(0.000)
log(l+0.05)	−1.498(0.008)	−1.451(0.008)	−1.710(0.008)
W×log(s)	−0.326(0.075)	−0.378(0.022)	0.500(0.211)
W×log(l+0.05)	0.574(0.497)	0.141(0.857)	2.150(0.211)
W×log(y)	0.742(0.000)	0.661(0.000)	0.883(0.000)
***Selection Criteria***			
Entropy	28.001	29.615	34.615
Bayesian	0.864	0.133	0.003
MJ	11.158	9.388	10.208
AIC	95.885	99.100	109.132

Note: *p*-values appear in parenthesis. Smaller values of *Entropy*, *MJ* and *AIC* criteria indicate a preferred **W**; larger values of the *Bayesian* posterior probability indicate a preferred **W**.

**Table 9:** Ertur & Koch case. Unrestricted SEM estimates.

*Model/Weight Matrix*	*SEM / W1*	*SEM / W2*	*SEM / W3*
constant	6.458(0.000)	6.706(0.000)	5.892(0.002)
$\log(s_{i})$	0.828(0.000)	0.804(0.000)	0.992(0.000)
$\log(l_{i}+0.05)$	−1.702(0.002)	−1.553(0.004)	−2.269(0.000)
$W\times \varepsilon_{i}$	0.823(0.000)	0.737(0.000)	0.937(0.000)
***Selection Criteria***			
Entropy	30.973	31.734	42.049
Bayesian	0.690	0.310	0.000
MJ	$0.171\times 10^{-12}$	$0.043\times 10^{-12}$	$0.085\times 10^{-12}$
AIC	97.870	99.391	120.021

Note: *p*-values appear in parenthesis. Smaller values of *Entropy*, *MJ* and *AIC* criteria indicate a preferred **W**; larger values of the *Bayesian* posterior probability indicate a preferred **W**.

## Appendix A

**Table A1:** Percentage of correct selections. DGP: SDM; Estimated equation SDM. T=1.

				CASE n = 25				CASE n = 49				CASE n = 100			
**T**	ρ	$\beta_{1}$	θ	**Entropy**	**Bayes**	**MJ**	**AIC**	**Entropy**	**Bayes**	**MJ**	**AIC**	**Entropy**	**Bayes**	**MJ**	**AIC**
1	−0.8	1	1	**70.40**	51.00	42.80	**70.40**	**89.10**	83.90	61.00	86.90	97.80	**98.00**	66.10	97.00
1	−0.5	1	1	45.30	17.50	37.30	**46.40**	**64.60**	34.90	48.60	62.60	**83.00**	69.80	54.70	81.80
1	−0.2	1	1	33.00	5.40	**37.00**	31.00	38.20	4.10	**45.30**	35.70	47.90	12.70	**48.50**	46.90
1	0.2	1	1	36.70	3.70	**40.80**	34.50	39.70	3.70	**42.40**	39.60	55.00	13.20	48.40	**55.50**
1	0.5	1	1	51.00	12.40	39.80	**52.60**	66.50	33.30	47.70	**69.70**	83.60	73.70	54.30	**87.70**
1	0.8	1	1	67.70	44.50	41.90	**72.60**	82.90	79.60	49.10	**86.50**	91.80	91.20	55.70	**95.30**
1	−0.8	1	5	81.20	61.60	62.60	**81.30**	**96.60**	95.50	85.80	95.90	99.90	**100.00**	98.20	99.60
1	−0.5	1	5	**66.90**	34.60	61.30	66.50	**89.80**	80.30	85.30	87.30	**98.80**	98.40	98.30	98.00
1	−0.2	1	5	**59.20**	19.60	64.10	56.30	80.70	53.40	**83.80**	77.70	**97.90**	96.20	97.80	96.60
1	0.2	1	5	62.70	16.00	**65.50**	60.50	79.40	46.10	**81.10**	77.50	96.20	92.60	**96.30**	96.20
1	0.5	1	5	70.33	29.17	62.74	**70.53**	88.00	69.60	79.60	**90.00**	98.20	97.30	96.20	**98.50**
1	0.8	1	5	79.00	58.40	57.10	**81.20**	93.20	89.80	75.10	**95.50**	99.40	99.40	92.10	**99.80**
1	−0.8	5	1	**73.90**	56.10	52.40	**73.90**	**92.40**	90.10	78.90	91.70	**99.60**	**99.60**	91.90	99.30
1	−0.5	5	1	50.00	19.50	42.50	**50.70**	**69.60**	42.40	57.60	68.00	**88.00**	80.90	69.70	85.60
1	−0.2	5	1	31.30	4.40	**35.50**	29.50	33.20	4.10	40.60	**33.10**	37.80	7.70	**38.50**	37.00
1	0.2	5	1	39.40	3.80	**46.10**	37.50	**48.70**	7.80	53.20	47.30	**70.00**	35.40	67.00	**70.00**
1	0.5	5	1	**59.10**	18.70	51.10	57.80	79.40	50.50	65.40	**81.10**	91.40	87.00	80.80	**93.70**
1	0.8	5	1	78.00	57.80	56.30	**80.70**	91.80	88.00	72.20	**94.40**	97.80	97.60	87.30	**99.10**
1	−0.8	5	5	67.70	50.40	44.30	**67.80**	**87.60**	84.90	64.50	86.80	97.90	**98.10**	73.00	97.40
1	−0.5	5	5	**53.00**	22.20	47.20	51.70	**76.70**	56.00	68.10	75.00	91.90	88.70	83.70	**90.20**
1	−0.2	5	5	53.90	12.80	**59.10**	49.70	75.70	41.30	**77.60**	71.90	93.80	88.30	**94.50**	92.00
1	0.2	5	5	66.00	20.60	**68.90**	62.70	86.20	60.90	**88.00**	84.40	98.10	96.10	97.70	**98.30**
1	0.5	5	5	**76.30**	42.30	71.40	76.10	95.30	82.60	90.50	**96.10**	99.70	99.50	99.60	**99.90**
1	0.8	5	5	88.00	73.30	73.60	**90.70**	97.70	96.30	91.10	**98.60**	**100.00**	99.90	99.40	**100.00**

**Table A2:** Percentage of correct selections. DGP: SDM; Estimated equation SDM. T=5.

Other Parameters				CASE n = 25				CASE n = 49				CASE n = 100			
**T**	ρ	$\beta_{1}$	θ	**Entropy**	**Bayes**	**MJ**	**AIC**	**Entropy**	**Bayes**	**MJ**	**AIC**	**Entropy**	**Bayes**	**MJ**	**AIC**
5	−0.8	1	1	97.30	**97.50**	47.40	97.10	**99.70**	99.80	59.60	99.60	**100.00**	**100.00**	61.00	**100.00**
5	−0.5	1	1	**76.10**	69.30	41.80	75.60	**100.00**	**100.00**	99.50	**100.00**	**100.00**	**100.00**	**100.00**	**100.00**
5	−0.2	1	1	40.30	21.80	**42.20**	36.40	**100.00**	**100.00**	95.80	**100.00**	**100.00**	**100.00**	99.90	**100.00**
5	0.2	1	1	57.90	45.90	53.30	**59.30**	**100.00**	99.90	70.70	99.80	**100.00**	**100.00**	80.80	**100.00**
5	0.5	1	1	79.00	78.30	56.20	**80.70**	92.60	**92.70**	49.50	91.80	99.30	**99.40**	59.90	98.90
5	0.8	1	1	93.10	93.50	59.70	**95.00**	99.80	**99.90**	99.20	99.80	**100.00**	**100.00**	**100.00**	**100.00**
5	−0.8	1	5	**99.90**	**99.90**	95.00	**99.90**	94.71	**95.20**	75.02	94.51	**100.00**	**100.00**	91.71	99.80
5	−0.5	1	5	**98.80**	98.70	96.30	98.40	98.30	**98.40**	90.50	97.70	**99.90**	99.90	99.20	99.70
5	−0.2	1	5	**97.00**	96.70	96.50	95.80	**60.30**	45.20	57.00	56.30	**80.80**	68.10	65.60	77.40
5	0.2	1	5	96.70	96.70	**97.00**	96.60	**99.40**	**99.40**	99.30	99.30	**100.00**	**100.00**	**100.00**	**100.00**
5	0.5	1	5	96.80	97.00	95.60	**97.40**	**49.40**	31.10	34.80	45.50	**66.60**	43.40	38.70	63.50
5	0.8	1	5	99.20	**99.30**	96.10	**99.30**	97.60	97.60	**97.70**	97.10	**100.00**	**100.00**	**100.00**	**100.00**
5	−0.8	5	1	99.00	**99.30**	88.10	98.70	72.20	65.30	64.40	**74.30**	86.50	83.40	80.30	**86.80**
5	−0.5	5	1	**82.80**	79.60	57.00	82.40	99.50	99.50	**99.60**	99.50	**100.00**	**100.00**	**100.00**	**100.00**
5	−0.2	5	1	**33.40**	14.00	32.00	30.20	83.30	81.90	80.00	**84.90**	95.30	95.00	93.90	**95.90**
5	0.2	5	1	**72.50**	65.30	70.20	**72.50**	99.80	99.80	**99.90**	**99.90**	**100.00**	**100.00**	**100.00**	**100.00**
5	0.5	5	1	91.30	**91.70**	85.50	91.40	90.70	90.90	68.10	**93.20**	98.40	98.60	81.20	**99.50**
5	0.8	5	1	98.90	98.80	92.90	**99.00**	**100.00**	**100.00**	99.40	**100.00**	**100.00**	**100.00**	**100.00**	**100.00**
5	−0.8	5	5	96.60	**97.10**	54.20	96.50	98.30	98.50	94.10	**99.00**	**100.00**	**100.00**	99.20	**100.00**
5	−0.5	5	5	**90.20**	89.60	77.40	90.00	**100.00**	**100.00**	**100.00**	**100.00**	**100.00**	**100.00**	**100.00**	**100.00**
5	−0.2	5	5	91.00	90.00	**92.30**	89.10	98.30	98.20	63.30	**99.40**	**100.00**	**100.00**	72.80	**100.00**
5	0.2	5	5	**98.00**	97.90	97.70	97.90	**100.00**	**100.00**	97.60	**100.00**	**100.00**	**100.00**	99.90	**100.00**
5	0.5	5	5	99.40	99.40	**99.80**	99.40	99.70	99.80	95.90	**100.00**	**100.00**	**100.00**	99.40	**100.00**
5	0.8	5	5	**99.90**	**99.90**	99.50	**99.90**	**100.00**	**100.00**	**100.00**	**100.00**	**100.00**	**100.00**	**100.00**	**100.00**

**Table A3:** Percentage of correct selections. DGP: SDM; Estimated equation SDM. T=10.

Other Parameters				CASE n = 25				CASE n = 49				CASE n = 100			
**T**	ρ	$\beta_{1}$	θ	**Entropy**	**Bayes**	**MJ**	**AIC**	**Entropy**	**Bayes**	**MJ**	**AIC**	**Entropy**	**Bayes**	**MJ**	**AIC**
10	−0.8	1	1	99.30	**99.40**	58.40	**99.30**	**100.00**	**100.00**	58.90	**100.00**	**100.00**	**100.00**	63.10	**100.00**
10	−0.5	1	1	**91.60**	89.70	49.90	91.50	**100.00**	**100.00**	**100.00**	**100.00**	**100.00**	**100.00**	**100.00**	**100.00**
10	−0.2	1	1	**59.80**	44.00	57.70	53.10	**100.00**	**100.00**	**100.00**	**100.00**	**100.00**	**100.00**	**100.00**	**100.00**
10	0.2	1	1	74.10	67.70	66.50	**76.40**	**100.00**	**100.00**	79.20	**100.00**	**100.00**	**100.00**	91.80	**100.00**
10	0.5	1	1	91.80	92.30	70.30	**94.10**	98.80	**99.10**	55.10	98.30	**100.00**	**100.00**	68.10	99.90
10	0.8	1	1	98.00	98.40	65.50	**98.90**	**100.00**	**100.00**	**100.00**	**100.00**	**100.00**	**100.00**	**100.00**	**100.00**
10	−0.8	1	5	**100.00**	**100.00**	**100.00**	**100.00**	**99.60**	**99.60**	89.80	99.40	**100.00**	**100.00**	98.20	**100.00**
10	−0.5	1	5	**100.00**	**100.00**	**100.00**	**100.00**	**99.90**	**99.90**	99.50	99.80	**100.00**	**100.00**	99.90	**100.00**
10	−0.2	1	5	**100.00**	**100.00**	99.90	**100.00**	**76.30**	64.90	64.20	71.00	**92.40**	90.40	80.50	90.40
10	0.2	1	5	**99.80**	**99.80**	**99.80**	**99.80**	**100.00**	**100.00**	**100.00**	**100.00**	**100.00**	**100.00**	**100.00**	**100.00**
10	0.5	1	5	99.80	99.80	99.50	**99.90**	**62.70**	43.70	37.20	59.00	**81.90**	73.70	40.70	80.50
10	0.8	1	5	**100.00**	99.90	99.00	**100.00**	**100.00**	**100.00**	**100.00**	**100.00**	**100.00**	**100.00**	**100.00**	**100.00**
10	−0.8	5	1	**100.00**	**100.00**	99.50	**100.00**	**87.20**	84.60	79.70	88.40	96.00	95.60	90.40	**96.50**
10	−0.5	5	1	95.30	**95.50**	81.90	94.50	**100.00**	**100.00**	**100.00**	**100.00**	**100.00**	**100.00**	**100.00**	**100.00**
10	−0.2	5	1	**48.40**	30.70	36.50	44.20	93.10	92.80	92.60	**93.80**	99.00	99.00	98.70	**99.30**
10	0.2	5	1	86.30	84.10	83.70	**87.20**	**100.00**	**100.00**	**100.00**	**100.00**	**100.00**	**100.00**	**100.00**	**100.00**
10	0.5	5	1	98.70	98.60	97.10	**99.00**	98.30	**98.80**	82.90	**98.80**	**99.90**	**99.90**	91.50	**99.90**
10	0.8	5	1	99.90	**100.00**	97.40	**100.00**	**100.00**	**100.00**	**100.00**	**100.00**	**100.00**	**100.00**	**100.00**	**100.00**
10	−0.8	5	5	**99.50**	**99.50**	74.60	99.40	**99.90**	**99.90**	99.60	**99.90**	**100.00**	**100.00**	**100.00**	**100.00**
10	−0.5	5	5	**98.90**	**98.90**	96.10	98.50	**100.00**	**100.00**	**100.00**	**100.00**	**100.00**	**100.00**	**100.00**	**100.00**
10	−0.2	5	5	98.90	99.00	**99.30**	99.00	99.90	**100.00**	74.90	**100.00**	**100.00**	**100.00**	76.30	**100.00**
10	0.2	5	5	**100.00**	**100.00**	**100.00**	**100.00**	**100.00**	**100.00**	**100.00**	**100.00**	**100.00**	**100.00**	**100.00**	**100.00**
10	0.5	5	5	**100.00**	**100.00**	**100.00**	**100.00**	**100.00**	**100.00**	99.80	**100.00**	**100.00**	**100.00**	**100.00**	**100.00**
10	0.8	5	5	**100.00**	**100.00**	99.90	**100.00**	**100.00**	**100.00**	**100.00**	**100.00**	**100.00**	**100.00**	**100.00**	**100.00**

**Table A4:** Percentage of correct selections. DGP: SDEM; Estimated equation SDEM. T=1.

				CASE n = 25				CASE n = 49				CASE n = 100			
**T**	ρ	$\beta_{1}$	θ	**Entropy**	**Bayes**	**MJ**	**AIC**	**Entropy**	**Bayes**	**MJ**	**AIC**	**Entropy**	**Bayes**	**MJ**	**AIC**
1	−0.8	1	1	54.30	50.70	31.00	**54.50**	67.40	64.50	30.50	**68.60**	80.30	80.50	28.20	**82.80**
1	−0.5	1	1	44.70	41.60	29.00	**45.80**	47.80	48.50	26.40	**52.50**	60.10	60.00	31.20	**65.30**
1	−0.2	1	1	36.40	32.50	27.50	**37.20**	32.50	28.80	28.70	**33.70**	33.00	26.60	31.00	**34.90**
1	0.2	1	1	**32.70**	25.30	30.80	30.40	26.60	17.40	**31.60**	24.80	35.10	17.90	**35.90**	34.10
1	0.5	1	1	34.20	26.30	**37.90**	32.10	44.10	35.90	**43.00**	42.20	**60.30**	55.80	37.10	58.80
1	0.8	1	1	42.80	40.50	**42.90**	39.50	61.60	**63.30**	47.80	53.90	78.10	**79.40**	44.00	67.70
1	−0.8	1	5	**60.30**	55.20	41.30	59.00	88.00	86.00	71.10	**89.20**	91.90	91.80	75.10	**92.70**
1	−0.5	1	5	56.20	52.20	44.80	**56.60**	**80.10**	77.80	73.70	**80.10**	85.20	85.20	77.60	**86.20**
1	−0.2	1	5	**51.60**	47.50	44.20	49.40	**68.10**	64.80	67.80	66.80	**77.00**	74.50	76.10	76.20
1	0.2	1	5	43.80	36.50	**43.90**	39.60	62.80	58.30	**66.20**	59.10	**75.00**	67.20	74.40	72.90
1	0.5	1	5	46.40	39.40	**47.20**	43.30	**66.60**	62.20	64.70	63.20	**81.00**	78.60	71.60	79.50
1	0.8	1	5	**54.20**	51.30	48.30	48.80	**75.90**	73.70	62.90	68.80	91.30	**91.60**	66.10	83.70
1	−0.8	5	1	**55.20**	50.90	31.90	54.40	70.60	67.50	27.80	**71.70**	83.80	83.10	27.90	**84.60**
1	−0.5	5	1	47.70	43.20	28.20	**47.80**	53.30	49.90	27.30	**58.50**	63.70	63.00	31.10	**67.40**
1	−0.2	5	1	35.30	28.80	29.10	**36.40**	34.10	29.50	27.80	**36.20**	34.50	24.40	28.90	**35.80**
1	0.2	5	1	28.10	19.20	**29.80**	27.60	**29.20**	17.90	34.70	28.40	34.00	16.20	**35.90**	32.40
1	0.5	5	1	32.50	23.80	**34.50**	29.60	**38.40**	30.00	36.10	37.50	**62.90**	56.10	39.40	61.10
1	0.8	5	1	**39.40**	35.60	37.50	35.90	57.70	**59.10**	46.70	49.40	79.60	**80.70**	42.90	69.10
1	−0.8	5	5	**63.20**	56.60	43.50	61.80	87.10	84.50	74.30	**87.70**	89.70	88.90	72.40	**90.60**
1	−0.5	5	5	**56.00**	49.30	41.80	55.30	78.50	73.50	71.40	**79.70**	84.10	83.20	74.70	**86.90**
1	−0.2	5	5	**49.00**	39.30	45.80	47.50	68.80	64.80	69.20	**70.20**	**76.20**	72.80	74.60	75.40
1	0.2	5	5	**44.70**	37.70	43.60	41.90	66.40	58.40	**68.50**	63.40	72.90	67.20	**73.40**	71.10
1	0.5	5	5	**44.80**	37.60	45.20	40.50	**67.50**	62.50	63.40	64.20	80.80	78.50	70.20	**81.10**
1	0.8	5	5	**48.50**	42.70	48.10	44.30	**71.50**	70.40	60.50	63.10	87.10	**87.60**	67.70	81.10

**Table A5:** Percentage of correct selections. DGP: SDEM; Estimated equation SDEM. T=5.

Other Parameters				CASE n = 25				CASE n = 49				CASE n = 100			
**T**	ρ	$\beta_{1}$	θ	**Entropy**	**Bayes**	**MJ**	**AIC**	**Entropy**	**Bayes**	**MJ**	**AIC**	**Entropy**	**Bayes**	**MJ**	**AIC**
5	−0.8	1	1	79.40	81.40	35.80	**82.80**	92.70	92.60	45.90	**94.20**	98.40	98.50	53.90	**99.20**
5	−0.5	1	1	48.40	**48.50**	32.20	47.70	74.50	74.90	49.00	75.50	90.20	90.90	56.60	**92.80**
5	−0.2	1	1	34.50	27.30	**37.10**	30.30	**51.00**	41.00	50.40	**46.60**	**60.70**	52.20	56.80	57.80
5	0.2	1	1	37.30	28.60	**38.70**	36.20	**53.50**	44.10	48.70	52.00	63.70	58.80	52.40	**64.00**
5	0.5	1	1	58.40	59.30	37.60	**59.50**	**78.70**	78.00	42.60	74.20	91.10	**92.70**	50.90	86.50
5	0.8	1	1	83.20	**83.30**	42.50	71.30	94.80	**95.50**	41.10	78.90	**99.00**	98.90	48.50	90.60
5	−0.8	1	5	97.70	97.70	93.50	**97.90**	99.80	99.80	98.90	**99.90**	**100.00**	**100.00**	99.80	**100.00**
5	−0.5	1	5	94.30	**95.10**	91.40	93.90	98.90	98.90	98.20	**99.10**	99.90	99.90	**100.00**	99.90
5	−0.2	1	5	89.90	89.10	**90.70**	87.00	**98.80**	98.70	98.50	98.50	**100.00**	**100.00**	**100.00**	**100.00**
5	0.2	1	5	**83.70**	82.60	83.60	83.10	**97.80**	97.70	**97.80**	97.40	**99.70**	**99.70**	**99.70**	99.50
5	0.5	1	5	**85.10**	84.80	79.10	83.60	**98.30**	**98.60**	95.60	96.80	**99.90**	**99.90**	99.20	99.70
5	0.8	1	5	**91.90**	91.70	70.70	82.80	99.50	**99.60**	88.00	93.50	**100.00**	**100.00**	96.10	98.60
5	−0.8	5	1	76.00	78.90	34.90	**79.80**	92.30	92.70	44.80	**94.20**	98.00	98.10	54.70	**98.60**
5	−0.5	5	1	52.15	51.85	34.57	**53.55**	75.92	75.62	48.45	**77.52**	91.71	92.91	57.74	**93.51**
5	−0.2	5	1	35.40	26.90	**36.70**	30.90	46.00	37.00	**48.30**	42.00	**62.30**	51.80	59.40	57.70
5	0.2	5	1	38.00	29.80	**38.20**	36.30	**51.00**	42.20	45.70	49.40	**69.20**	62.10	56.10	68.10
5	0.5	5	1	**60.60**	59.10	40.30	59.10	76.10	**77.20**	42.90	72.30	90.00	**91.10**	49.30	85.90
5	0.8	5	1	83.40	**83.50**	41.70	69.90	95.10	**95.80**	40.30	77.10	98.40	**98.60**	46.80	90.70
5	−0.8	5	5	**97.90**	97.80	93.20	**97.90**	99.60	99.60	98.60	**99.70**	**100.00**	**100.00**	**100.00**	**100.00**
5	−0.5	5	5	**94.20**	**94.20**	92.10	92.50	99.00	99.10	98.40	**99.30**	99.90	99.90	99.90	**100.00**
5	−0.2	5	5	90.30	89.10	**90.50**	88.00	99.10	**99.30**	98.80	98.90	**99.80**	**99.80**	99.50	**99.80**
5	0.2	5	5	83.70	82.70	**84.20**	83.40	98.20	**98.40**	98.10	97.50	99.80	**99.90**	99.70	99.80
5	0.5	5	5	**84.40**	83.70	80.20	81.20	97.50	**97.70**	95.00	96.30	**99.70**	**99.70**	98.90	99.40
5	0.8	5	5	91.80	**92.00**	70.90	82.80	**99.00**	**99.00**	87.10	94.80	**100.00**	**100.00**	95.10	99.00

**Table A6:** Percentage of correct selections. DGP: SDEM; Estimated equation SDEM. T=10.

Other Parameters				CASE n = 25				CASE n = 49				CASE n = 100			
**T**	ρ	$\beta_{1}$	θ	**Entropy**	**Bayes**	**MJ**	**AIC**	**Entropy**	**Bayes**	**MJ**	**AIC**	**Entropy**	**Bayes**	**MJ**	**AIC**
10	−0.8	1	1	89.90	90.30	43.00	**91.00**	98.80	98.80	59.30	**99.20**	99.90	99.90	70.80	**100.00**
10	−0.5	1	1	**66.50**	63.40	43.00	63.50	**85.60**	85.30	56.30	85.80	97.10	**97.70**	72.10	97.60
10	−0.2	1	1	**49.00**	39.00	47.20	42.60	**59.50**	49.60	59.20	53.20	**79.10**	74.10	71.70	73.30
10	0.2	1	1	**48.60**	43.90	41.90	**48.60**	**63.80**	60.30	53.80	**63.80**	80.80	**80.90**	69.30	80.80
10	0.5	1	1	78.00	**78.90**	45.60	75.30	90.10	**90.80**	50.60	84.50	96.10	**96.80**	62.10	93.00
10	0.8	1	1	**96.80**	96.60	42.80	83.20	99.20	**99.50**	45.10	88.00	**100.00**	**100.00**	51.90	97.10
10	−0.8	1	5	99.60	**99.70**	98.50	**99.70**	**100.00**	**100.00**	99.90	**100.00**	**100.00**	**100.00**	**100.00**	**100.00**
10	−0.5	1	5	98.00	98.10	97.20	**98.30**	**99.90**	**99.90**	**99.90**	**99.90**	**100.00**	**100.00**	**100.00**	**100.00**
10	−0.2	1	5	**97.50**	**97.50**	97.30	97.40	**100.00**	**100.00**	99.80	**100.00**	**100.00**	**100.00**	**100.00**	**100.00**
10	0.2	1	5	95.30	95.20	95.50	**95.70**	**99.70**	**99.70**	**99.70**	**99.70**	**100.00**	**100.00**	**100.00**	**100.00**
10	0.5	1	5	**97.30**	**97.30**	93.00	96.60	**99.40**	**99.40**	**99.30**	**99.20**	**100.00**	**100.00**	**100.00**	**100.00**
10	0.8	1	5	**98.90**	98.70	83.90	92.90	**100.00**	99.90	95.20	99.00	**100.00**	**100.00**	99.80	**100.00**
10	−0.8	5	1	90.70	**91.40**	42.60	92.40	98.00	97.60	55.00	**98.70**	99.70	**99.80**	70.60	**99.80**
10	−0.5	5	1	**65.80**	62.60	45.50	61.30	**87.80**	87.20	56.70	87.30	96.60	**96.80**	70.70	**96.80**
10	−0.2	5	1	**47.80**	37.30	44.40	42.90	**60.20**	50.30	56.70	53.30	**78.40**	74.60	71.30	74.70
10	0.2	5	1	**47.00**	41.60	42.80	45.00	**65.90**	61.10	52.30	64.40	**79.30**	78.40	66.10	76.80
10	0.5	5	1	76.30	**76.50**	45.60	72.60	90.80	**90.90**	50.40	84.90	97.00	**97.20**	64.20	93.70
10	0.8	5	1	96.20	**97.00**	40.50	82.40	99.30	**99.40**	44.60	88.70	**99.80**	**99.80**	51.50	95.90
10	−0.8	5	5	**99.70**	**99.70**	97.60	**99.70**	**100.00**	**100.00**	**100.00**	**100.00**	**100.00**	**100.00**	**100.00**	**100.00**
10	−0.5	5	5	98.80	98.90	97.60	**99.10**	**99.90**	**99.90**	**99.90**	**99.90**	**100.00**	**100.00**	**100.00**	**100.00**
10	−0.2	5	5	97.60	**97.90**	97.70	97.30	**100.00**	**100.00**	**100.00**	**100.00**	**100.00**	**100.00**	**100.00**	**100.00**
10	0.2	5	5	96.60	96.50	**96.70**	96.30	99.90	99.90	99.90	**100.00**	**100.00**	**100.00**	**100.00**	**100.00**
10	0.5	5	5	**97.00**	**97.00**	93.70	95.20	**100.00**	**100.00**	99.50	99.50	**100.00**	**100.00**	99.90	**100.00**
10	0.8	5	5	**99.00**	**99.00**	83.90	93.50	99.80	**99.90**	96.10	98.80	**100.00**	**100.00**	99.60	**100.00**

**Table A7:** Percentage of correct selections. DGP: SLM; Estimated equation SLM. T=1.

			CASE n = 25				CASE n = 49				CASE n = 100			
**T**	ρ	$\beta_{1}$	**Entropy**	**Bayes**	**MJ**	**AIC**	**Entropy**	**Bayes**	**MJ**	**AIC**	**Entropy**	**Bayes**	**MJ**	**AIC**
1	−0.8	1	**70.80**	70.70	50.80	70.70	**88.80**	84.70	45.40	86.90	**98.20**	98.10	59.40	97.50
1	−0.5	1	45.00	41.70	42.90	**46.50**	**63.70**	39.40	40.20	62.90	**81.80**	67.80	46.60	79.30
1	−0.2	1	22.90	15.10	**33.20**	24.10	29.90	6.60	**35.50**	30.50	**39.30**	6.60	35.70	38.90
1	0.2	1	29.50	11.10	**35.00**	28.60	36.10	3.00	**40.20**	33.20	47.40	6.00	33.80	**48.90**
1	0.5	1	**50.80**	36.20	38.40	51.20	65.60	32.40	39.90	69.00	79.30	66.40	41.10	**83.80**
1	0.8	1	68.80	66.30	49.50	**71.20**	81.50	77.20	42.30	**86.80**	91.40	90.20	46.60	**95.00**
1	−0.8	5	85.20	**86.40**	73.70	84.70	**96.50**	96.10	84.30	94.80	**99.80**	**99.80**	94.20	99.60
1	−0.5	5	**58.30**	56.10	50.20	56.60	**75.40**	59.40	62.10	73.20	**91.60**	87.80	80.60	89.30
1	−0.2	5	26.20	14.40	**30.60**	23.40	35.40	7.30	**42.10**	33.30	50.40	14.70	**52.10**	48.70
1	0.2	5	**50.80**	27.70	43.10	49.90	39.60	5.80	**40.90**	39.30	**53.20**	10.70	48.60	53.10
1	0.5	5	**70.60**	67.90	62.20	70.20	71.60	46.40	57.20	**75.40**	85.90	77.40	70.50	**89.20**
1	0.8	5	79.40	**80.00**	71.30	77.50	88.30	83.00	62.80	**92.70**	96.60	96.40	82.60	**98.20**

**Table A8:** Percentage of correct selections. DGP: SLM; Estimated equation SLM. T=5.

			CASE n = 25				CASE n = 49				CASE n = 100			
**T**	ρ	$\beta_{1}$	**Entropy**	**Bayes**	**MJ**	**AIC**	**Entropy**	**Bayes**	**MJ**	**AIC**	**Entropy**	**Bayes**	**MJ**	**AIC**
5	−0.8	1	97.10	**98.10**	57.20	96.70	**100.00**	**100.00**	69.60	99.90	**100.00**	**100.00**	81.80	**100.00**
5	−0.5	1	80.40	**81.10**	43.20	80.40	**91.50**	91.90	50.80	90.30	**99.50**	**99.50**	61.60	98.80
5	−0.2	1	39.30	29.10	**39.40**	37.80	**48.90**	27.70	37.70	45.20	**69.80**	42.40	43.70	67.60
5	0.2	1	50.10	40.70	32.00	**51.60**	59.70	39.70	34.80	**63.20**	77.40	57.60	33.40	**80.60**
5	0.5	1	79.80	80.80	35.20	**82.90**	89.10	88.50	40.40	**92.30**	96.10	96.40	44.30	**97.80**
5	0.8	1	92.10	92.80	38.40	**93.80**	98.60	98.10	50.90	**99.40**	99.90	**100.00**	48.10	**100.00**
5	−0.8	5	**99.80**	**99.80**	**98.80**	99.70	**100.00**	**100.00**	99.90	**100.00**	**100.00**	**100.00**	**100.00**	**100.00**
5	−0.5	5	91.90	**92.60**	79.20	90.90	**98.60**	**98.60**	94.80	98.30	**100.00**	**100.00**	99.70	**100.00**
5	−0.2	5	**51.00**	44.50	43.40	47.60	66.30	49.70	**66.70**	59.40	**87.00**	75.10	81.60	83.60
5	0.2	5	63.70	58.50	43.90	**63.90**	72.70	60.30	61.10	**74.50**	85.00	76.20	72.50	**85.50**
5	0.5	5	87.30	88.20	63.90	**90.00**	97.20	97.40	91.40	**98.40**	99.80	99.80	98.20	**99.90**
5	0.8	5	97.50	97.40	71.40	**97.90**	**99.90**	**100.00**	96.10	**100.00**	**100.00**	**100.00**	98.30	**100.00**

**Table A9:** Percentage of correct selections. DGP: SLM; Estimated equation SLM. T=10.

			CASE n = 25				CASE n = 49				CASE n = 100			
**T**	ρ	$\beta_{1}$	**Entropy**	**Bayes**	**MJ**	**AIC**	**Entropy**	**Bayes**	**MJ**	**AIC**	**Entropy**	**Bayes**	**MJ**	**AIC**
1	−0.8	1	99.50	**99.60**	66.00	99.40	**100.00**	**100.00**	70.60	**100.00**	**100.00**	**100.00**	89.90	**100.00**
1	−0.5	1	93.00	**93.80**	50.10	92.70	98.20	**98.60**	56.00	98.10	99.80	**99.90**	72.80	99.70
1	−0.2	1	**53.80**	47.80	34.60	50.60	**62.80**	45.60	37.00	58.80	**84.00**	75.30	44.60	81.50
1	0.2	1	62.04	58.54	34.07	**63.44**	70.00	60.80	32.10	**73.90**	86.31	83.12	40.76	**87.61**
1	0.5	1	88.40	89.50	41.90	**90.10**	95.60	96.20	45.30	**97.70**	99.60	**99.80**	57.90	**99.80**
1	0.8	1	97.90	97.90	44.70	**98.60**	99.80	99.60	50.10	**99.90**	**100.00**	**100.00**	54.80	**100.00**
1	−0.8	5	**100.00**	**100.00**	99.90	**100.00**	**100.00**	**100.00**	99.90	**100.00**	**100.00**	**100.00**	**100.00**	**100.00**
1	−0.5	5	98.60	**98.80**	94.70	97.90	**99.80**	**99.80**	98.20	99.60	**100.00**	**100.00**	**100.00**	**100.00**
1	−0.2	5	**68.50**	66.60	54.40	65.40	**78.30**	71.20	70.50	73.80	**95.40**	94.70	89.00	94.30
1	0.2	5	**73.10**	72.50	51.70	73.00	83.40	80.00	73.40	**83.60**	95.40	94.90	86.90	**96.20**
1	0.5	5	97.10	**97.40**	81.50	97.00	99.30	**99.40**	95.80	**99.40**	**100.00**	99.90	99.80	**100.00**
1	0.8	5	99.80	**99.90**	84.90	99.80	**100.00**	**100.00**	98.20	**100.00**	**100.00**	**100.00**	99.80	**100.00**

**Table A10:** Percentage of correct selections. DGP: SDEM; Estimated equation SDM. T=1.

				CASE n = 25				CASE n = 49				CASE n = 100			
**T**	ρ	$\beta_{1}$	θ	**Entropy**	**Bayes**	**MJ**	**AIC**	**Entropy**	**Bayes**	**MJ**	**AIC**	**Entropy**	**Bayes**	**MJ**	**AIC**
1	−0.8	1	1	58.70	23.40	30.80	**59.10**	**67.00**	40.90	37.60	66.90	79.80	66.70	34.10	**81.80**
1	−0.5	1	1	47.00	11.60	29.50	**49.20**	47.30	18.70	36.60	**50.10**	62.10	33.00	34.90	**66.40**
1	−0.2	1	1	37.50	6.60	28.30	**39.10**	32.80	6.70	35.30	**33.50**	37.00	6.60	**39.60**	38.70
1	0.2	1	1	26.50	4.20	**32.20**	26.80	26.70	3.70	**40.10**	24.10	36.80	3.50	**38.50**	36.40
1	0.5	1	1	25.50	6.10	**35.30**	24.00	38.40	16.20	**43.70**	35.90	**61.80**	39.30	41.30	59.70
1	0.8	1	1	34.90	17.50	**36.80**	32.70	**57.40**	50.50	47.50	50.60	77.30	**80.20**	46.00	66.10
1	−0.8	1	5	59.70	32.10	41.30	**62.20**	74.30	52.50	54.20	**77.90**	88.80	79.00	62.50	**92.40**
1	-0.5	1	5	54.90	25.80	44.10	**55.80**	**64.00**	40.40	55.30	63.70	82.80	62.00	69.70	**83.70**
1	−0.2	1	5	**50.20**	19.60	46.70	48.30	60.10	31.30	**63.10**	56.20	75.00	46.50	**76.90**	72.00
1	0.2	1	5	42.80	12.30	**44.10**	40.10	58.00	27.80	**64.60**	52.70	77.40	47.40	**78.40**	74.30
1	0.5	1	5	39.40	12.60	**45.30**	36.30	**61.30**	38.60	60.70	55.70	**84.10**	72.30	78.30	79.50
1	0.8	1	5	44.30	21.80	**44.90**	38.10	**65.80**	60.10	53.70	59.60	87.70	**90.40**	51.60	77.50
1	−0.8	5	1	**58.10**	20.50	35.50	**58.10**	67.40	39.50	41.00	**70.60**	80.90	69.40	36.30	**84.70**
1	−0.5	5	1	47.10	14.60	32.60	**48.40**	47.50	19.60	36.20	**50.40**	63.50	31.70	34.80	**67.50**
1	−0.2	5	1	38.70	8.20	32.10	**39.80**	30.70	7.10	**36.60**	32.10	37.40	7.30	37.50	**37.80**
1	0.2	5	1	26.70	4.90	**32.10**	26.80	29.40	3.00	**41.10**	26.60	**43.80**	4.40	43.20	40.90
1	0.5	5	1	28.50	5.50	**34.40**	26.30	39.10	17.80	**43.80**	36.90	**59.80**	42.40	46.20	56.10
1	0.8	5	1	33.90	17.80	**37.10**	31.60	**57.20**	53.70	48.40	49.90	78.00	**81.10**	46.20	67.80
1	−0.8	5	5	61.80	31.30	43.00	**63.70**	75.00	54.60	54.00	**79.50**	89.30	79.20	62.10	**91.10**
1	−0.5	5	5	57.40	24.30	42.40	**58.50**	69.70	42.20	59.10	**70.30**	79.50	60.60	69.70	**81.40**
1	−0.2	5	5	45.80	19.10	43.70	**47.00**	62.20	34.60	**64.20**	60.40	72.60	43.50	**74.60**	70.40
1	0.2	5	5	42.80	11.50	**45.60**	41.40	57.80	27.40	**61.60**	53.50	78.00	47.40	**79.10**	75.00
1	0.5	5	5	42.90	10.80	**45.90**	38.60	**60.70**	37.70	59.20	56.70	**83.40**	74.50	77.10	80.40
1	0.8	5	5	**44.10**	24.20	43.80	38.80	**64.10**	61.40	53.40	56.40	86.80	**88.90**	52.90	77.20

**Table A11:** Percentage of correct selections. DGP: SDEM; Estimated equation SDM. T=5.

Other Parameters				CASE n = 25				CASE n = 49				CASE n = 100			
**T**	ρ	$\beta_{1}$	θ	**Entropy**	**Bayes**	**MJ**	**AIC**	**Entropy**	**Bayes**	**MJ**	**AIC**	**Entropy**	**Bayes**	**MJ**	**AIC**
5	−0.8	1	1	76.90	68.10	37.50	**81.10**	92.40	90.90	37.80	**94.10**	97.10	97.30	48.70	**98.70**
5	−0.5	1	1	48.90	34.50	36.40	**49.60**	71.00	55.30	39.00	**73.20**	90.70	89.40	52.70	**92.40**
5	−0.2	1	1	33.10	15.00	**38.10**	28.20	38.20	17.50	**41.70**	34.10	**56.70**	41.90	53.50	51.80
5	0.2	1	1	37.40	21.20	**38.50**	36.50	**48.50**	28.80	41.90	47.70	**64.40**	60.30	55.40	64.00
5	0.5	1	1	**60.00**	58.10	42.40	59.60	**76.40**	76.00	42.30	72.70	90.90	**92.10**	47.60	87.10
5	0.8	1	1	86.60	**87.20**	46.60	71.20	94.60	**95.30**	51.90	78.00	98.80	**99.00**	47.90	87.40
5	-0.8	1	5	**87.50**	83.70	74.30	86.30	98.30	96.50	83.60	**98.90**	**100.00**	**100.00**	99.70	**100.00**
5	−0.5	1	5	**79.50**	74.30	78.00	72.00	**94.00**	90.60	87.40	92.70	**99.80**	**99.80**	**99.80**	**99.80**
5	−0.2	1	5	81.80	77.10	**85.20**	75.40	89.50	84.30	**90.20**	84.50	**99.70**	**99.70**	**99.70**	**99.70**
5	0.2	1	5	82.10	79.40	**83.70**	78.00	**92.80**	90.90	92.60	90.20	99.20	99.20	**99.30**	98.40
5	0.5	1	5	81.20	**81.40**	76.70	75.90	91.20	**92.70**	83.30	83.20	97.80	**98.00**	90.20	94.80
5	0.8	1	5	85.30	**88.80**	50.10	69.30	93.60	**94.70**	57.50	75.80	99.00	**99.10**	20.50	90.60
5	−0.8	5	1	77.20	68.40	38.70	**80.50**	92.10	89.40	40.00	**93.80**	98.30	98.30	49.40	**99.00**
5	−0.5	5	1	**49.35**	33.97	37.66	48.55	73.23	54.85	40.36	**73.63**	89.31	87.41	50.25	**91.51**
5	−0.2	5	1	**33.80**	17.10	37.50	30.20	**39.40**	15.00	**39.40**	34.60	**56.20**	41.70	**56.20**	51.60
5	0.2	5	1	36.70	22.00	**38.10**	35.90	**48.60**	27.50	42.10	47.90	62.90	60.40	54.20	**64.50**
5	0.5	5	1	**57.20**	55.10	40.40	56.90	**76.80**	76.40	43.10	71.40	88.90	**90.50**	50.10	83.80
5	0.8	5	1	**86.00**	85.80	48.70	71.90	94.40	**95.50**	50.30	78.30	99.00	**99.10**	47.60	89.60
5	−0.8	5	5	**85.90**	82.50	72.40	84.40	97.50	96.70	83.00	**98.00**	**100.00**	**100.00**	99.20	**100.00**
5	−0.5	5	5	**81.80**	76.00	80.60	75.00	**93.60**	90.90	88.70	91.70	**100.00**	**100.00**	**100.00**	**100.00**
5	−0.2	5	5	82.60	77.10	**84.50**	75.60	90.90	85.90	**92.60**	85.90	**99.80**	**99.80**	**99.80**	**99.80**
5	0.2	5	5	**84.20**	82.40	85.30	81.80	90.60	89.80	**90.80**	88.60	98.80	99.10	**99.30**	98.10
5	0.5	5	5	84.70	**86.60**	77.30	78.80	89.10	**91.30**	83.30	81.30	98.20	98.50	91.80	**93.40**
5	0.8	5	5	88.00	**89.20**	50.00	73.20	93.50	**94.40**	57.90	75.70	99.30	**99.40**	20.30	91.00

**Table A12:** Percentage of correct selections. DGP: SDEM; Estimated equation SDM. T=10.

Other Parameters				CASE n = 25				CASE n = 49				CASE n = 100			
**T**	ρ	$\beta_{1}$	θ	**Entropy**	**Bayes**	**MJ**	**AIC**	**Entropy**	**Bayes**	**MJ**	**AIC**	**Entropy**	**Bayes**	**MJ**	**AIC**
10	−0.8	1	1	89.10	84.50	40.40	**90.90**	**97.90**	97.40	49.90	98.70	**99.90**	**99.90**	58.90	**99.90**
10	−0.5	1	1	**65.00**	53.10	42.40	58.50	**87.40**	81.10	53.60	86.70	**97.30**	96.90	62.20	**97.60**
10	−0.2	1	1	41.50	29.40	**42.40**	37.00	**56.80**	39.80	54.50	50.80	**75.90**	66.70	69.60	71.80
10	0.2	1	1	**49.40**	41.80	43.50	48.00	66.40	59.90	55.70	**66.20**	**77.90**	76.20	66.70	76.00
10	0.5	1	1	74.60	**75.20**	42.60	72.50	87.40	**88.60**	51.10	82.80	96.30	**97.00**	58.50	92.00
10	0.8	1	1	**96.60**	96.40	42.80	78.90	99.00	**99.20**	48.00	88.30	99.90	**100.00**	47.00	95.10
10	−0.8	1	5	**91.50**	89.30	75.90	87.20	**100.00**	**100.00**	99.40	**100.00**	**100.00**	**100.00**	**100.00**	**100.00**
10	−0.5	1	5	**89.20**	87.00	87.10	77.60	99.90	99.90	**100.00**	99.90	**100.00**	**100.00**	**100.00**	**100.00**
10	−0.2	1	5	93.50	90.90	**94.40**	83.70	99.60	99.60	**99.90**	99.40	**100.00**	**100.00**	**100.00**	**100.00**
10	0.2	1	5	96.80	**97.30**	96.80	95.30	99.10	**99.30**	99.10	98.70	99.90	99.90	**100.00**	99.80
10	0.5	1	5	93.60	**94.80**	87.80	85.80	98.70	**99.00**	94.80	94.30	**99.70**	**99.70**	98.60	98.50
10	0.8	1	5	95.60	**96.70**	36.80	76.70	**99.90**	99.60	32.40	88.30	**100.00**	**100.00**	18.50	94.40
10	−0.8	5	1	88.60	83.60	41.40	**89.50**	97.90	97.40	52.00	**98.70**	99.70	99.80	62.80	**99.90**
10	-0.5	5	1	**64.70**	52.60	43.60	58.90	**84.70**	78.50	53.50	84.60	96.80	95.90	61.90	**97.30**
10	−0.2	5	1	**45.50**	31.00	44.50	38.40	**57.20**	42.20	53.40	51.90	**76.20**	64.60	66.70	70.80
10	0.2	5	1	**50.10**	43.30	45.60	48.70	**66.00**	57.80	58.70	64.30	**79.60**	78.80	65.30	78.00
10	0.5	5	1	74.70	**75.50**	45.20	72.30	89.80	**91.30**	50.40	84.30	96.10	**96.80**	59.30	93.10
10	0.8	5	1	97.00	**97.10**	43.50	83.10	99.30	**99.60**	48.20	86.20	**100.00**	**100.00**	51.00	96.10
10	−0.8	5	5	**90.40**	88.20	73.70	86.50	99.90	99.90	98.90	**100.00**	**100.00**	**100.00**	**100.00**	**100.00**
10	−0.5	5	5	**89.50**	87.40	86.90	77.60	**99.90**	**99.90**	99.60	99.80	**100.00**	**100.00**	**100.00**	**100.00**
10	−0.2	5	5	93.60	91.30	**93.90**	84.90	99.70	99.70	**99.80**	99.50	**100.00**	**100.00**	**100.00**	**100.00**
10	0.2	5	5	95.40	95.60	**96.00**	93.60	99.50	**99.70**	**99.70**	99.40	**100.00**	**100.00**	**100.00**	99.90
10	0.5	5	5	93.60	**95.40**	88.00	87.70	98.60	**98.80**	94.50	94.20	99.60	**99.70**	99.00	98.70
10	0.8	5	5	95.40	**97.20**	33.00	76.20	99.30	**99.40**	34.00	88.30	99.70	**99.90**	18.10	95.90
